# Drop-off-reinitiation triggered by EF-G-driven mistranslocation and its alleviation by EF-P

**DOI:** 10.1093/nar/gkac068

**Published:** 2022-02-21

**Authors:** Kenya Tajima, Takayuki Katoh, Hiroaki Suga

**Affiliations:** Department of Chemistry, Graduate School of Science, The University of Tokyo, 7-3-1 Hongo, Bunkyo-ku, Tokyo 113-0033, Japan; Department of Chemistry, Graduate School of Science, The University of Tokyo, 7-3-1 Hongo, Bunkyo-ku, Tokyo 113-0033, Japan; Department of Chemistry, Graduate School of Science, The University of Tokyo, 7-3-1 Hongo, Bunkyo-ku, Tokyo 113-0033, Japan

## Abstract

In ribosomal translation, peptidyl transfer occurs between P-site peptidyl-tRNA and A-site aminoacyl-tRNA, followed by translocation of the resulting P-site deacylated-tRNA and A-site peptidyl-tRNA to E and P site, respectively, mediated by EF-G. Here, we report that mistranslocation of P-site peptidyl-tRNA and A-site aminoacyl-tRNA toward E and A site occurs when high concentration of EF-G triggers the migration of two tRNAs prior to completion of peptidyl transfer. Consecutive incorporation of less reactive amino acids, such as Pro and d-Ala, makes peptidyl transfer inefficient and thus induces the mistranslocation event. Consequently, the E-site peptidyl-tRNA drops off from ribosome to give a truncated peptide lacking the C-terminal region. The P-site aminoacyl-tRNA allows for reinitiation of translation upon accommodation of a new aminoacyl-tRNA at A site, leading to synthesis of a truncated peptide lacking the N-terminal region, which we call the ‘reinitiated peptide’. We also revealed that such a drop-off-reinitiation event can be alleviated by EF-P that promotes peptidyl transfer of Pro. Moreover, this event takes place both *in vitro* and in cell, showing that reinitiated peptides during protein synthesis could be accumulated in this pathway in cells.

## INTRODUCTION

In ribosomal translation, nascent polypeptide chains are elongated by repeating the following three fundamental steps: (i) peptidyl transfer from the P-site peptidyl-tRNA onto the A-site aminoacyl-tRNA catalyzed by the peptidyl transferase center of ribosome, (ii) translocation of the resulting P-site deacylated-tRNA and A-site peptidyl-tRNA to the E site and P site, respectively, mediated by EF-G and (iii) accommodation of a new aminoacyl-tRNA onto the empty A site mediated by EF-Tu (1). Although the P-site peptidyl-tRNA is indispensable for the peptidyl transfer reaction, the peptidyl-tRNA can be lost under certain conditions by drop-off from the ribosome, leading to truncation of peptides (2–5). For instance, elongation of inefficient substrates such as consecutive l-proline (Pro) residues (6–9) or some kinds of nonproteinogenic amino acids, e.g. β-, d- and *N*-methyl-amino acids, often suffers from peptidyl-tRNA drop-off (5). Moreover, since peptidyl-tRNA is stabilized at the P-site by interaction with the ribosomal exit tunnel, ones with short nascent peptides are more prone to drop-off due to insufficient interaction with the tunnel. Thus, drop-off is promoted by some macrolide antibiotics that hinder the interaction between the nascent peptide and the exit tunnel (10–14).

The dropped peptidyl-tRNA is eventually hydrolyzed by peptidyl-tRNA hydrolase (PTH) at the ester bond between peptide and 2′/3′-hydroxyl group of tRNA (15), ending up with generation of a truncated peptide lacking the C-terminal region, which we refer to as ‘drop-off peptide (DoP)’. On the other hand, the fate of the ribosome that has lost the peptidyl-tRNA is unclear due to the lack of comprehensive study of such a complex. Given the aminoacyl-tRNA as well as the mRNA still remain in ribosome, it is possible that the translation reinitiates by the migration of the aminoacyl-tRNA from A site to P site along with the mRNA, followed by accommodation of a new aminoacyl-tRNA onto the empty A site. In that case, a truncated peptide lacking the N-terminal region, which we call ‘reinitiated peptide (RiP)’, could be generated utilizing the remaining aminoacyl-tRNA as an N-terminal building block. Indeed, two related papers published by our group indicated such a possibility that not only peptidyl-tRNA drop-off but also RiP synthesis could occur (5,16). However, the mechanism how drop-off-reinitiation occurs to generate such RiPs remains elusive.

As for the translocation of the remaining aminoacyl-tRNA from A site to P site, EF-G would be the possible candidate responsible for such an event, similar to the case with the canonical translocation (17). Based on the fact that EF-G directly interacts with only A-site tRNA (18), we assumed that EF-G moves the A-site aminoacyl-tRNA toward the P site, and thereby the P-site peptidyl-tRNA is pushed out toward E site and eventually drops off, which we refer to as a ‘mistranslocation’ event. In order to verify this hypothesis, we have monitored mistranslocation events by increasing the EF-G concentration in expression of model peptides containing inefficient substrates, Pro or d-Ala, in a consecutive manner. As it has been previously reported that EF-P improves the efficiency of peptidyl transfer between consecutive Pro residues (7,8), the effect of EF-P on mistranslocation was also evaluated. In addition, the effect of length and sequence of nascent peptides, Shine-Dalgarno (SD)-like sequences in ORF, and other types of possible drop-off inducers such as RF2, RF3 and RRF are also evaluated.

## MATERIALS AND METHODS

### Preparation of cDNA templates, mRNA templates, Flexizymes and tRNAs

cDNA templates, mRNA templates, Flexizymes (dFx and eFx) and tRNAs used for translation and pre-charging of activated amino acids were prepared by PCR and *in vitro* transcription (see Supplementary Table S1A–H for their sequences). Template DNAs were prepared by extension of forward and reverse primer pairs, followed by PCR using forward and reverse PCR primers. The PCR products were extracted by phenol/chloroform, precipitated by ethanol, and used for transcription at 37°C for at least 3 h in a 100–2000 μl reaction mixture. For mRNA and Flexizymes, reaction mixture contained 40 mM tris(hydroxymethyl)aminomethane (Tris)–HCl (pH 8.0), 1 mM spermidine, 0.01% Triton X-100, 10 mM DTT, 30 mM MgCl_2_, 5 mM NTPs, 30 mM KOH, 10% template DNA solution and 0.12 μM T7 RNA polymerase. For tRNAs, reaction mixture contained 40 mM Tris–HCl (pH 8.0), 1 mM spermidine, 0.01% Triton X-100, 10 mM DTT, 22.5 mM MgCl_2_, 3.75 mM NTPs, 5 mM GMP, 22.5 mM KOH, 10% template DNA solution and 0.12 μM T7 RNA polymerase. The resulting RNA transcripts were treated with RQ1 DNase (Promega) for 1 h at 37°C and purified by 8% polyacrylamide gel containing 6 M urea.

### Aminoacylation of tRNAs

Activated amino acids [l-lysine-3,5-dinitrobenzyl ester (Lys-DBE), l-asparagine-3,5-dinitrobenzyl ester (Asn-DBE), l-glutamate-3,5-dinitrobenzyl ester (Glu-DBE), l-leucine-3,5-dinitrobenzyl ester (Leu-DBE), l-isoleucine-3,5-dinitrobenzyl ester (Ile-DBE), l-phenylalanine-cyanomethyl ester (Phe-CME), l-methionine-3,5-dinitrobenzyl ester (Met-DBE), *N*-biotinylated l-phenylalanine-cyanomethyl ester (^Bio^F-CME) and d-alanine-3,5-dinitrobenzyl ester (d-Ala-DBE)] were synthesized by previously reported methods (19,20). Aminoacylation was carried out at 0°C in a reaction mixture containing 50 mM HEPES–KOH (pH 7.5), 600 mM MgCl_2_, 20% DMSO, 25 μM dFx or eFx, 25 μM tRNA and 5 mM activated amino acids. eFx was used for aminoacylation of Phe-CME and ^Bio^F-CME and dFx for that of other amino acids. Reaction time was 2 h for all the activated amino acids. The aminoacyl-tRNAs were recovered by ethanol precipitation, and then pellet was washed twice with 70% ethanol containing 0.1 M sodium acetate (pH 5.2), once with 70% ethanol and dissolved in 1 mM sodium acetate (pH 5.2).

### Preparation of EF-P for *in vitro* translation reactions

*Escherichia coli efp* gene was cloned into a modified pET28a(+) vector that has PreScission protease recognition site instead of thrombin site (Supplementary Table S1I). *E. coli epmA* and *epmB* genes were cloned into pETDuet-1 vector. These vectors were co-introduced into *E. coli* Rosetta2 (DE3) pLysS. The cells were cultured in LB medium with 0.5 mM IPTG for 2 h at 37°C and lysed by sonication. The cell lysate was applied to HiTrap TALON crude column (Cytiva) to purify the histidine-tagged EF-P. The column was washed with buffer A (20 mM Tris–HCl (pH 8.0), 200 mM NaCl, 2 mM imidazole and 1 mM 2-mercaptoethanol) and then the histidine-tagged EF-P was eluted by buffer A containing 500 mM imidazole. Turbo3C protease was added to the eluate for cleaving the histidine-tag and dialyzed against the buffer A at 4°C overnight. The sample was applied on the HiTrap TALON crude column, and the flow-through and wash fractions were recovered as EF-P without histidine-tag. Then, the protein was concentrated by Amicon Ultra 10k centrifugal filter (Merck Millipore).

### Expression of peptides in the reconstituted cell-free translation system

Peptides were expressed by utilizing modified FIT (Flexible *in vitro* translation system), in which unnecessary amino acids, aminoacyl-tRNA synthetases (aaRSs) and EF-P were withdrawn from the following mixture (See also supplementary Table S2 for the details of the translation conditions). The complete FIT system contains 2 μM template mRNA, 0.5 mM each 20 proteinogenic l-amino acids (Ala, Cys, Asp, Glu, Phe, Gly, His, Ile, Lys, Leu, Met, Asn, Pro, Gln, Arg, Ser, Thr, Val, Trp, Tyr), 2 mM ATP, 2 mM GTP, 1 mM CTP, 1 mM UTP, 20 mM creatine phosphate, 50 mM HEPES–KOH (pH 7.6), 100 mM potassium acetate, 12.8 mM magnesium acetate, 2 mM spermidine, 1 mM DTT, 1 mM tris(2-carboxyethyl)phosphine (TCEP), 0.1 mM 10-HCO-H4folate, 1.5 mg/ml *E. coli* tRNAs, 1.2 μM *E. coli* ribosome, 0.6 μM MTF, 2.7 μM IF1, 0.4 μM IF2, 1.5 μM IF3, 0.26 μM EF-G, 10 μM EF-Tu, 0.66 μM EF-Ts, 5 μM EF-P, 0.25 μM RF2, 0.17 μM RF3, 0.5 μM RRF, 4 μg/ml creatine kinase, 3 μg/ml myokinase, 0.1 μM inorganic pyrophosphatase, 0.1 μM T7 RNA polymerase, 0.73 μM AlaRS, 0.03 μM ArgRS, 0.38 μM AsnRS, 0.13 μM AspRS, 0.02 μM CysRS, 0.06 μM GlnRS, 0.23 μM GluRS, 0.09 μM GlyRS, 0.02 μM HisRS, 0.4 μM IleRS, 0.04 μM LeuRS, 0.11 μM LysRS, 0.03 μM MetRS, 0.68 μM PheRS, 0.16 μM ProRS, 0.04 μM SerRS, 0.09 μM ThrRS, 0.03 μM TrpRS, 0.02 μM TryRS and 0.02 μM ValRS. The concentration of EF-G was changed if necessary. To incorporate two consecutive d-Ala into a peptide, the FLPS2a peptide was expressed from mRNAS2 in the modified FIT system containing 50 μM d-Ala-tRNA^GluE2^_CGG_, only five amino acids (0.5 mM [^12^C]-Asp or 0.05 mM [^14^C]-Asp, 0.5 mM Gly, 0.5 mM Lys, 0.5 mM Met and 0.5 mM Tyr) and five aaRSs (0.13 μM AspRS, 0.09 μM GlyRS, 0.11 μM LysRS, 0.03 μM MetRS and 0.02 μM TryRS). To distinguish whether drop-off is caused by mRNA context or nascent peptides, peptides were expressed from mRNAs utilizing the corresponding pre-charged aminoacyl-tRNAs in the modified FIT system containing only three amino acids (0.5 mM [^12^C]-Asp or 0.05 mM [^14^C]-Asp, 0.5 mM Met and 0.5 mM Tyr) and three aaRSs (0.13 μM AspRS, 0.03 μM MetRS and 0.02 μM TryRS). FLP3 was expressed from mRNA3 with 25 μM Leu-tRNA^AsnE2^_GAG_ and 50 μM Lys-tRNA^AsnE2^_CUU_, or from mRNA4 with 25 μM Leu-tRNA^AsnE2^_CUU_ and 50 μM Lys-tRNA^AsnE2^_GAG_. FLP2 was expressed from mRNA2 with 50 μM Lys-tRNA^AsnE2^_CUU_, or mRNA3 with 25 μM Lys-tRNA^AsnE2^_GAG_ and 50 μM Lys-tRNA^AsnE2^_CUU_. FLP4 was expressed from mRNA5 with 25 μM Asn-tRNA^AsnE2^_GUU_, 50 μM Lys-tRNA^AsnE2^_CUU_ and 25 μM Glu-tRNA^AsnE2^_CUC_, or from mRNA6 with 25 μM Asn-tRNA^AsnE2^_GAG_, 25 μM Lys-tRNA^AsnE2^_GAU_, 25 μM Glu-tRNA^AsnE2^_GAA_ and 50 μM Lys-tRNA^AsnE2^_CUU_. FLP5 was expressed from mRNA6 with 25 μM Leu-tRNA^AsnE2^_GAG_, 25 μM Ile-tRNA^AsnE2^_GAU_, 25 μM Phe-tRNA^AsnE2^_GAA_ and 50 μM Lys-tRNA^AsnE2^_CUU_, or from mRNA7 with 25 μM Leu-tRNA^AsnE2^_GUU_, 25 μM Ile-tRNA^AsnE2^_CUU_, 25 μM Phe-tRNA^AsnE2^_CUC_ and 50 μM Lys-tRNA^AsnE2^_GAG_. In coding different amino acids in the initiation and elongation AUG codons, FLPM1(^Bio^F, M) was expressed from mRNAM1 with 100 μM ^Bio^F-tRNA^fMet^_CAU_ and 50 μM Met-tRNA^AsnE2^_CAU_ without Met, MetRS and 10-HCO-H4folate or with 100 μM ^Bio^F-tRNA^fMet^_CAU_, 0.5 mM Met and MetRS without 10-HCO-H4folate. In expression of representative peptides with various kinds of nascent peptide sequences prior to the three consecutive prolines, peptides were expressed with 12.5 μM ^Bio^F-tRNA^fMet^_CAU_ without 10-HCO-H4folate. The reaction mixture was incubated at 37°C for 20 min. In the identification of dropped peptidyl-tRNA or its nascent chain, the translation reaction mixture was incubated with 10 μM kanamycin (Nacalai tesque) for 5 min at 37°C, followed by incubation with 1 μM PTH for 10 min at 37°C, if necessary. In separation of the dropped peptidyl-tRNA and the peptidyl-tRNA trapped in the ribosome, the reaction mixture was mixed with equivalent volume of MeOH, centrifuged for 3 min at 13 000 rpm, and the supernatant and the precipitation was separated. The precipitation was re-resolved into 1× FIT buffer (20 mM Tris–HCl (pH 7.6), 50 mM KCl and 1 mM DTT), mixed with f.c. 12.8 mM EDTA, f.c. 10 μM kanamycin, f.c. 15.8 mM Mg(OAc)_2_, f.c. 1 μM PTH and incubated at 37°C for 10, 30 or 60 min. Finally, the reaction mixture was incubated with f.c. 50 μg/ml of RNase A (Funakoshi) for 30 min at 37°C if necessary.

In the identification by MALDI-TOF MS, the translation products were purified by anti-FLAG antibody if necessary. After addition of equal amount of 2× TBS buffer (100 mM Tris–HCl (pH 7.6) and 300 mM NaCl), the reaction mixture was incubated with anti-FLAG antibody agarose gel (Sigma) for 1 h at room temperature, followed by wash with 1× TBS buffer (50 mM Tris–HCl (pH 7.6) and 150 mM NaCl) twice and eluted with 0.1% TFA solution. Reaction mixture and elution were desalted with SPE C-tip (Nikkyo Technos) and eluted with 1.2 μl of 80% acetonitrile and 0.5% acetic acid solution containing 50% saturated (*R*)-cyano-4-hydroxycinnamic acid (Bruker Daltonics). MALDI-TOF MS analysis was performed using ultrafleXtreme (Bruker Daltonics) in reflector positive mode. Peptide calibration standard II (Bruker Daltonics) was used for the external mass calibration.

For quantification of peptide yields, 0.5 mM [^12^C]-Asp, [^12^C]-Lys or [^32^S]-Met in the FIT system were replaced with radio-labeled 0.05 mM [^14^C]-Asp, 0.05 mM [^14^C]-Lys or 0.868 μM [^35^S]-Met. After incubation, reaction mixture was mixed with equal volume of 2x tricine-SDS-PAGE loading solution (0.9 M Tris–HCl, 8w/v% SDS, 30% glycerol, 0.001w/v% xylene cyanol, pH 8.45) and incubated at 95°C for 5 min. The solution was applied to the tricine-SDS polyacrylamide gel composed of 4% stacking gel and 15% separation gel and run for 40 min at 150 V with cathode buffer (0.1 mM Tris–HCl, 0.1 mM tricine, and 0.1w/v% SDS, pH 8.45) and anode buffer (0.2 mM Tris–HCl pH 8.9). After drying, the gel was exposed to an imaging plate for overnight and analyzed by Typhoon FLA 7000 (Cytiva).

In the identification and quantification by LC-ESI MS, proteins were precipitated by incubation of the reaction solution with equal volume of methanol on ice for 5 min, centrifuged at 13 000 rpm for 3 min, and centrifuged with 4× volume of 1% TFA at 13 000 rpm for 3 min. The resulting solution was injected to the LC-ESI MS (Xevo™ G2-XS, Waters) assembled with Acquity UPLC BEH C18 column (1.7 μm, 300Å, 2.1 × 150 mm, Waters) and eluted by gradient of water/acetonitrile containing 0.1% formic acid.

### Profiling of nascent peptide sequence against peptidyl-tRNA drop-off and RiP generation

Nascent chain-dependent Pro/Gly incorporation efficiency was analyzed by an mRNA display-based profiling system. 1.2 μM mRNA conjugated with puromycin was translated in 2.5 μl of FIT system in the presence of 12.5 μM of ^Bio^F-tRNA^fMet^_CAU_ and in the absence of 10-HCO-H4folate, with 0.03, 0.26 or 10 μM EF-G and in the presence or absence of 5 μM EF-P. The reaction mixture was incubated at 37°C for 10 min, followed by incubation with f.c. 16.7 mM EDTA at 37°C for 5 min and reverse transcription by adding 1.72 μl of RT solution [0.73 mM dNTPs, 73.4 mM Tris–HCl (pH 8.3), 4.9 μM reverse primer, 29 mM KOH, 43.7 mM Mg(OAc)_2_ and 14.6 U/μl RTase (Promega)] at 42°C for 1 h. The reaction was quenched by addition of 0.5 μl of 100 mM EDTA and 0.55 μl of 0.2 M HCl. 0.5 μl of reaction mixture was aliquoted as ‘Initial’ fraction, diluted into 1× PCR mixture [50 mM KCl, 10 mM Tris–HCl (pH 9.0), 0.1% Triton X-100, 2.5 mM MgCl_2_, 0.25 mM dNTPs and 0.25 μM of forward and reverse primer] by 100-fold. Remaining solution was incubated at room temperature for 10 min with 2.5 μl slurry of Dynabeads M-280 streptavidin (Veritas) washed by 1× TBST buffer (50 mM Tris–HCl (pH 7.6), 150 mM NaCl and 0.05% Tween-20) three times. After removal of supernatant, the Dynabeads was washed with 100 μl of 1× TBST and transferred to the new tube 10 times. The cDNA of ^Bio^F-peptide-mRNA/cDNA conjugates were eluted by 1× PCR mix by incubation at 95°C for 5 min as ‘Pull-down’ fraction. After addition of Taq DNA polymerase into 1× PCR mixture, the amount of cDNA in the ‘Initial’ fraction and ‘Pull-down’ fraction was quantified by qPCR and amplified by PCR in optimum thermal cycles. Amplified cDNAs were purified by phenol/chloroform extraction and ethanol precipitation. Adopter sequences for next generation sequencing were added to the purified cDNAs by PCR and purified by NucleoSpin Gel and PCR Clean-up (Macherey-Nagel). The purified cDNAs were sequenced by Miseq and Miseq reagent kit v3 (150 cycles) (Illumina). The ratio of a nascent peptide chain n in the ‘Initial’ or ‘Pull-down’ fraction, *P_n(Ini)_* and *P_n(PD__−__/+)_*, enrichment of n in the presence or absence of EF-P through the selection process, *E_n-/+_*_,_ and incorporation enhancement by EF-P, *W_n_* were calculated as following equations;

$$\begin{eqnarray*}{\rm{\ }}{P_{n\left( {Ini} \right)}} &=& \frac{{Coun{t_{n\left( {Ini} \right)}}}}{{\mathop \sum \nolimits_n^{8420} Coun{t_{n\left( {Ini} \right)}}}}\ \\ {P_{n\left( {PD - / + } \right)}} &=& \frac{{Coun{t_{n\left( {PD - / + } \right)}}}}{{\mathop \sum \nolimits_n^{8420} Coun{t_{n\left( {PD - / + } \right)}}}}\ \end{eqnarray*}$$

$$\begin{equation*}{\rm{\ }}{E_{n - / + }} = {\rm{lo}}{{\rm{g}}_2}\ \left( {\frac{{{P_{n\left( {PD - / + } \right)}}}}{{{P_{n\left( {Ini} \right)}}}}} \right),\ \ \ {W_n} = {E_{n + }}{\rm{\ }} - {E_{n - }}\end{equation*}$$

### Preparation of plasmid coding YhhM and *efp* under an arabinose promoter

pBAD-P_BAD_-YhhM-His_6_ plasmid was prepared as follows (Supplementary Table S1I). cDNAs of YhhM (purchased from Integrated DNA Technologies) were amplified by adopter PCR in order to add 15 nt homologous regions and purified by NucleoSpin Gel and PCR Clean-up (Macherey-Nagel). pBAD-HisA ColE1 ori plasmid (Thermo Fisher Invitrogen) were amplified with the same homologous region as those added to the cDNA of proteins by inversion PCR. PCR reaction contained 50% KODone premix (TOYOBO), 0.3 μM forward and reverse primers, and 1.00 ng/μl linear or circular template DNA. PCR reaction was performed at 94°C for 120 s, followed by repeat of 94°C for 10 s, 56°C for 30 s and 68°C for 60 s/1 kb. DNAs were purified by 1w/v% agarose gel electrophoresis. The resulting cDNA and linear plasmids were assembled by In-Fusion HD cloning kit (Takara bio). Transformation was carried out with 50 μl *E. coli* DH5α competent cells with 2.5 μl of In-Fusion reaction mixture, incubated on ice for 10 min, heat-shocked for 45 s at 42°C, incubated on ice for 2 min, and spread on LB plates containing 100 μg/ml ampicillin at 37°C for overnight. Single colonies were picked and cultivated in 4 ml LB broth with 100 μg/ml ampicillin at 37°C for overnight, and then plasmid was extracted by fast gene plasmid mini kit (Nippon Gene) and sequenced (Fasmaq). pBAD-P_BAD_-YhhM-fh plasmid was prepared in the same procedure shown above.

pBAD-*efp*-p15A ori plasmid was prepared as follows. First, pBAD-*efp-*ColE1 ori plasmid was prepared by In-Fusion assembly (Takara bio) of an inversion PCR product of pBAD-HisA ColE1 ori plasmid (Thermo Fisher Invitrogen) and PCR product of *efp* gene from pET28a(+)-*efp* plasmid. Transformation was carried out with 50 μl *E. coli* DH5α competent cells with 2.5 μl of In-Fusion reaction mixture, following the same procedure as preparation of pBAD-P_BAD_-YhhM-His_6_ plasmid using ampicillin. Then a DNA fragment containing *araC*-P_BAD_ promotor-*efp* was amplified by PCR of the pBAD-*efp*-ColE1 ori plasmid and a DNA fragment containing CmR-p15A ori was amplified by inversion PCR from pLysS plasmid, which were assembled by In-Fusion reaction. Transformation was carried out with 50 μl *E. coli* DH5α competent cells with 2.5 μl In-Fusion reaction mixture, following the same procedure as preparation of pBAD-P_BAD_-YhhM-His_6_ plasmid using 30 μg/ml chloramphenicol in LB. The recombinant plasmid was prepared and sequenced following the procedure shown above.

### Complementation of *E. coli* cells lacking chromosomal *efp* gene by pBAD-efp-p15A ori plasmid

Expression and post-translational modification of EF-P was confirmed by using *E. coli* JW4107 strain lacking its chromosomal *efp* gene (*E. coli* Δ*efp*) (National BioResource Project-*E. coli*). *E. coli* Δ*efp* was transformed with pBAD-*efp*-p15A ori plasmid. The resulting *E. coli* Δ*efp*/pBAD-*efp*-p15A ori (*E. coli* Δ*efp*/+*efp*) was cultivated in 0.5 L LB with 50 μg/ml kanamycin and 30 μg/ml chloramphenicol for 4.5 h at 37°C and expression of *efp* gene was induced by 0.02% l-arabinose for 4 h at 37°C. The *E. coli* pellet was recovered by centrifugation at 8000 rpm for 10 min, flash-frozen and stored at −80°C. The pellet was resuspended with lysis buffer (20 mM Tris–HCl (pH 7.4), 200 mM NaCl, 1 mM DTT and 0.1 mM PMSF), sonicated and centrifuged at 15 000 g for 10 min. The supernatant was filtered by Minisart NML GF and Minisart NML hydrophile (Sartorius) and applied to HiTrap TALON crude (Cytiva). Then, EF-P was eluted with imidazole gradient and digested by 100 ng/μl Glu-C proteinase (Sigma) in 50 mM Tris–HCl (pH 8.0). After methanol precipitation and trifluoroacetic acid precipitation, the solution was analyzed by LC-ESI MS (Xevo^TM^ G2-XS, Waters) assembled with Acquity UPLC BEH C18 column (1.7 μm, 300Å, 2.1 × 150 mm, Waters) and eluted by gradient of water/acetonitrile containing 0.1% formic acid.

### Expression and purification of YhhM-fh in *E. coli* Δ*efp* and *E. coli* Δ*efp*/+*efp*

After transformation of *E. coli* Δ*efp* strain and *E. coli* Δ*efp*/+*efp* stain by pBAD-P_BAD_-YhhM-fh plasmid, the resulting single colony was pre-cultivated in 2 ml of LB with 100 μg/ml ampicillin and 50 μg/ml of kanamycin for the *E. coli* Δ*efp* stain and with additional 30 μg/ml chloramphenicol for *E. coli* Δ*efp*/+*efp* stain at 37°C for overnight. The preculture broth was added to 100 ml of LB broth containing the same antibiotics, cultivated at 37°C for 4 h, and induced with f.c. 0.02% l-arabinose at 37°C for 4 h. *E. coli* pellet was recovered by centrifugation at 8000 rpm for 10 min, flash-frozen and stored at −80°C. The pellet was resuspended with lysis buffer (20 mM Tris–HCl (pH 7.4), 200 mM NaCl, 1 mM DTT and 0.1 mM PMSF), sonicated and centrifuged at 15 000 g for 10 min. The supernatant was applied to pre-equilibrated 1 ml slurry of His Super Flow (Cytiva) and eluted by imidazole gradient. The fractions containing YhhM-fh were analyzed by glycine-SDS-PAGE. The band corresponding to YhhM-fh was eluted from the gel and purified by PD10 (Cytiva) in order to remove DTT. Then, YhhM-fh was further purified by anti-FLAG M2 agarose gel (Sigma) and concentrated by Amicon Ultra 10k MW cut-off (Merck Millipore).

### Sequence identification of YhhM-fh digested with Lys-N by LC-ESI MS/MS

0.67 μg of YhhM-fh expressed in *E. coli* Δ*efp* and 1.3 μg of YhhM-fh expressed in *E. coli* Δ*efp*/+*efp* was digested by 0.027 μg/μl Lys-N (Thermo Fisher Invitrogen) with 0.05 M NH_4_HCO_3_ (pH 8.2) in 30 μl reaction mixture at 37°C for 16 h. The reaction mixtures were desalted by SPE C-tip (Nikkyo Technos) and eluted with 30 μl of 80% acetonitrile with 0.5% acetic acid solution. The eluates were analyzed by LC-ESI MS^E^ and LC-ESI MS/MS (Xevo™ G2-XS, Waters) assembled with Acquity UPLC BEH C18 column (1.7 μm, 300 Å, 2.1 × 150 mm, Waters) and eluted by gradient of water/acetonitrile containing 0.1% formic acid. First, the parent ions were screened by MS^E^ (Data-Independent Acquisition, DIA) method. *m/z* values corresponding to parent peptide fragments were screened by UniFi software (Waters). In order to sequence the N-terminal fragments, parent ions were fragmented by MS/MS mode. In MS/MS analysis, parent ions having a specific *m/z* value was selected by the quadrupole and fragmented by collision-induced dissociation using ramping of collision energy from 10 to 40 V. The daughter ions were identified by MassLynx software (Waters) after deconvolution of raw MS/MS spectra.

## RESULTS

### Peptidyl-tRNA drop-off and reinitiated peptide synthesis upon incorporation of consecutive Pro residues

**Figure 1.**
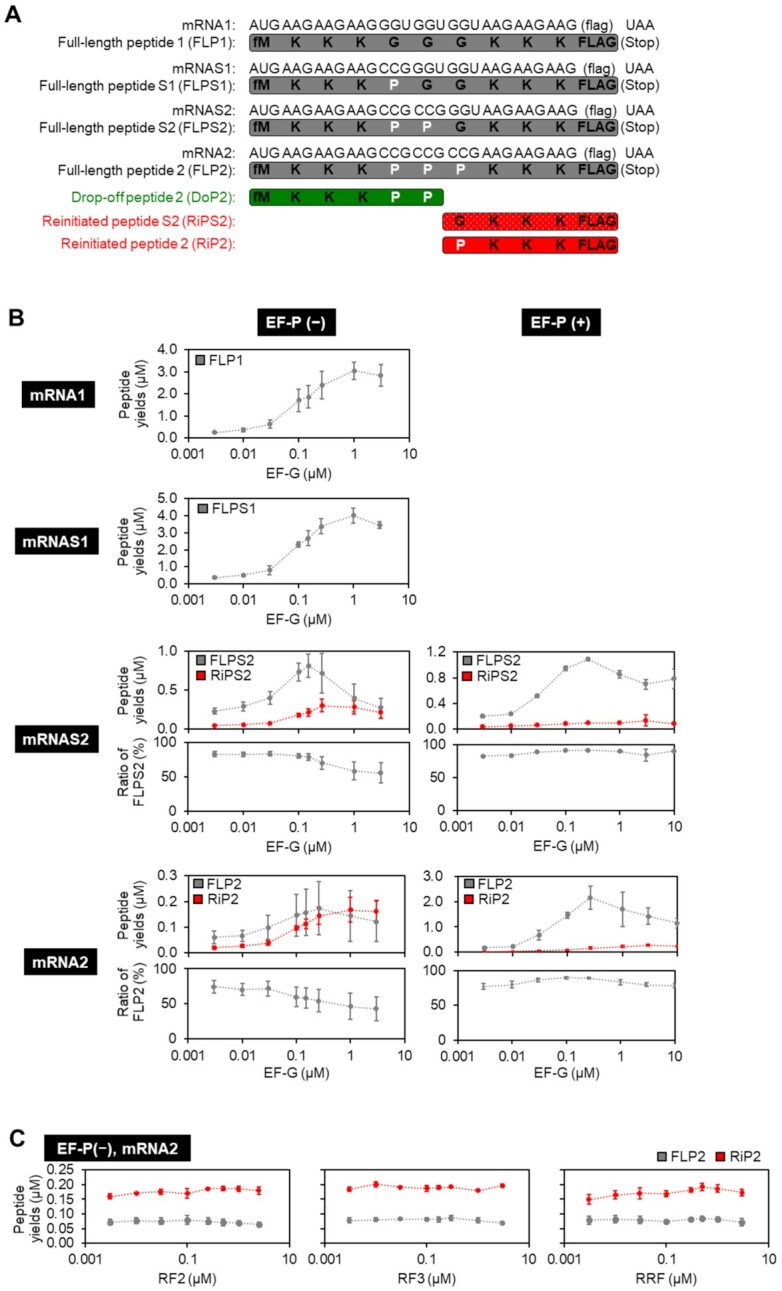
Quantification of full-length and reinitiated peptides in incorporation of consecutive Pro residues. (**A**) mRNA and expressed peptide sequences: full-length peptide (FLP1, FLPS1, FLPS2 and FLP2), drop-off peptide (DoP2), and reinitiated peptide (RiP2). (**B**) Titration of EF-G in translation of mRNA1, mRNAS1, mRNAS2 and mRNA2 in the absence or presence of 5 μM EF-P. Expressed peptides were labelled by [^14^C]-Asp and quantified by autoradiography. Expression levels of FLP1, FLPS1, FLPS2, FLP2, RiPS2 and RiP2 are shown. The ratio of FLPS2 or FLP2 was defined as (the yield of FLPS2 or FLP2)/(the sum of yields of translation products). Since only FLPs were observed in translation of mRNA1 and mRNAS1, ratio of them were omitted. *n* = 3. (**C**) Titration of RF2, RF3 and RRF in translation of mRNA2 in the absence of EF-P. Yields of FLP2 and RiP2 were quantified by autoradiography. *n* = 3.

**Figure 2.**
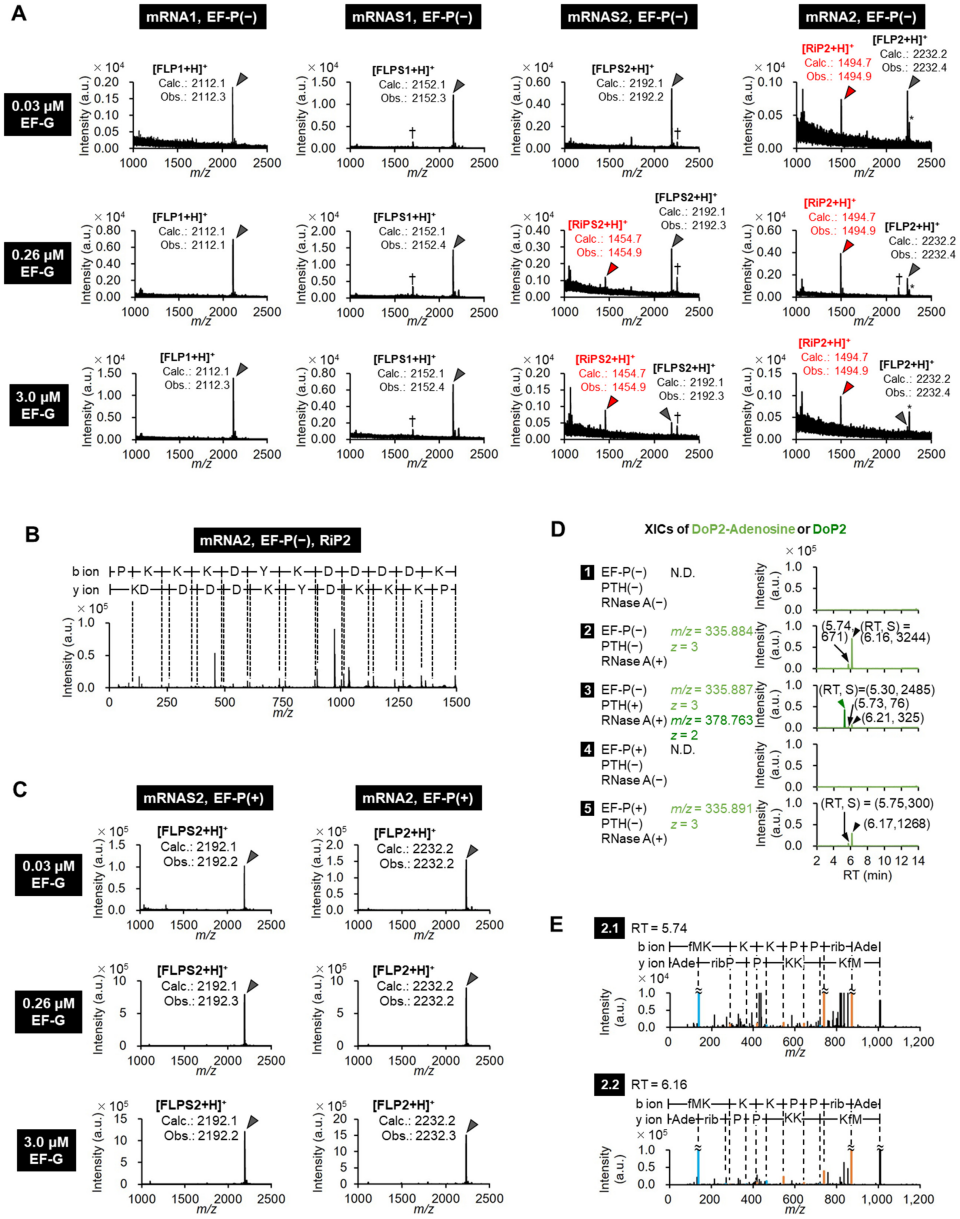
Mass spectrometric analysis of full-length and truncated peptides in incorporation of consecutive Pro. (**A**) Identification of translation products by MALDI-TOF MS. Peptides were expressed with 0.03, 0.26 or 3.0 μM EF-G in the absence of EF-P. *: Na^+^ or K^+^ adduct peaks, †: Unidentified peaks. ‘Calc.’ and ‘Obs.’ indicate calculated and observed *m/z* values, respectively. (**B**) MALDI-TOF MS/MS identification of RiP2 generated in the translation of mRNA2 with 0.26 μM EF-G. (**C**) Identification of translation products expressed in the presence of 5 μM EF-P by MALDI-TOF MS. (**D**) LC-ESI MS identification of drop-off DoP2-tRNA^Pro^ derivatives treated with PTH and/or RNase A. Their extracted ion current chromatograms (XICs) were extracted by using the indicated *m*/*z* values. Concentrations of EF-P, PTH and RNase A were 5 μM, 1 μM and 50 μg/ml, respectively. Black arrow-heads indicate the peaks of DoP2-Adenosine, and a green arrow-head DoP2. **(E)** Deconvoluted LC-ESI MS/MS spectra of DoP2-Adenosine observed at RT = 5.74 min (2.1) and RT = 6.16 min (2.2) in the XIC 2 in Figure 2D.

To observe the EF-G-driven mistranslocation event, we conducted *in vitro* translation of mRNAs into model peptides containing consecutive Pro residues with titration of EF-G concentration. mRNA2 encodes a model full-length peptide FLP2 that have three consecutive Pro, whereas mRNA1 encodes a negative control peptide FLP1 that has three Gly in place of Pro (Figure 1A). Since peptidyl transfer between consecutive Pro is extremely inefficient due to the poor abilities in peptidyl donor and acceptor (9,21,22), we expected to observe drop-off-reinitiation in translation of mRNA2 but not in mRNA1. Peptides were radioisotope-labelled by incorporating [^14^C]-aspartic acid (Asp, D) in the C-terminal FLAG sequence (Asp-Tyr-Lys-Asp-Asp-Asp-Asp-Lys) for quantification of product bands in tricine SDS-PAGE by autoradiography. In translation of mRNA1, only the full-length product FLP1 was observed [Figure 1B, Supplementary Figure S1A: mRNA1, EF-P (−)], whereas two products were observed in mRNA2: the full-length FLP2 and the reinitiated peptide RiP2 derived from truncation between the second and third Pro residues [Figure 1B, Supplementary Figure S1A: mRNA2, EF-P (−), grey and red, respectively]. To further confirm the identity of these peptides, mRNA1 and mRNA2 were translated with [^12^C]-cold Asp instead of [^14^C]-Asp, and analyzed by MALDI-TOF mass spectrometry (MS) and MALDI-TOF MS/MS, in which only FLP1 was detected in the translation of mRNA1, whereas both FLP2 and RiP2 were detected in the translation of mRNA2 (Figure 2A, B: mRNA1 and mRNA2). The drop-off peptide DoP2 was not detected because PTH is not added in this analysis and thus DoP2 should not be released from the tRNA. These results indicate that the mistranslocation event occurs with fMet-Lys-Lys-Lys-Pro-Pro-tRNA at P site and Pro-tRNA at A site; the former dropped off and the latter migrated to P site, then translation is reinitiated from the Pro-tRNA to generate the RiP2.

In order to analyze the effect of the number of consecutive Pro residues on mistranslocation, translation of mRNAS1 and mRNAS2, expressing the full-length FLPS1 and FLPS2 containing one and two consecutive Pro residues, respectively, was conducted, and the products were compared to those of mRNA1 and mRNA2 (Figures 1A, B, 2A, Supplementary Figure S1A). An intense peak of RiPS2 along with FLPS2 were observed for mRNAS2, similar to the products of RiP2 and FLP2 expressed from the mRNA2 template. On the other hand, the mRNAS1 template produced only the full-length peptide FLPS1. This indicates that two consecutive incorporations of Pro is less efficient than its single incorporation, promoting the RiP synthesis. In the case of mRNAS2 translation, the expression level of the full-length FLPS2 dramatically decreased at higher concentrations of EF-G [Figure 1B: mRNAS2, EF-P (−)], indicating that EF-G indeed triggers peptidyl-tRNA drop-off and thereby inhibits the usual level of FLPS2 synthesis. The expression level of RiPS2 increased at such high concentrations of EF-G, indicating that EF-G mediates the migration of A-site aminoacyl-tRNA to P site for the reinitiation. Consequently, the ratio of the RiPs increased in an EF-G concentration-dependent manner. A similar tendency was also observed in the translation of mRNA2, in which the ratio of RiP2 increases at higher concentrations of EF-G [Figure 1B: mRNA2, EF-P (−)]. These results show that both inefficient peptidyl transfer (i.e. consecutive Pro) and frequent translocation (i.e. high EF-G concentration) lead to the drop-off-reinitiation event. Since not only EF-G but also RF2, RF3 and RRF have been previously suggested to induce peptidyl-tRNA drop-off (23–25), we also titrated them in the translation of mRNA2; however, no significant enhancement in the RiP2 synthesis was observed with higher concentration of those factors (Figure 1C). Thus, we concluded that RF2, RF3 and RRF were not involved in the drop-off-reinitiation event.

Next, we analyzed the effect of EF-P on the drop-off-reinitiation. It has been previously reported that EF-P recognizes a specific D-arm motif found in the tRNA^Pro^ isoacceptors and accelerates peptidyl transfer in the elongation of Pro (7,8,26). EF-P is also involved in alleviation of translocation defects arising from miscoding and frameshifting (27,28). In translation of mRNA2, EF-P greatly enhanced the expression level of FLP2 and reduced that of RiP2 (Figure 1B: mRNA2, 2.2 μM of FLP2 and 0.1 μM RiP2 in the presence of EF-P and 0.26 μM EF-G, while 0.17 μM of FLP2 and 0.14 μM RiP2 in the absence of EF-P and presence of 0.26 μM EF-G), showing that efficient peptidyl transfer could suppress the drop-off-reinitiation. Since EF-P requires β-lysinylation at Lys34 for its full activity, we also evaluated the effect of using an unmodified EF-P in translation of mRNA2 into FLP2 and RiP2 (Supplementary Figure S2). Consequently, even the unmodified EF-P could suppress RiP synthesis and enhance the ratio of FLP, although its effect was weaker than the use of β-lysinylated EF-P. This result indicates that the EF-P in the E site blocks the movement of the P-site peptidyl-tRNA to the E site, thereby suppressing peptidyl-tRNA drop-off. Both the acceleration of peptidyl transfer and the blocking of the movement of the P-site tRNA should contribute to the suppression of drop-off-reinitiation event.

**Figure 3.**
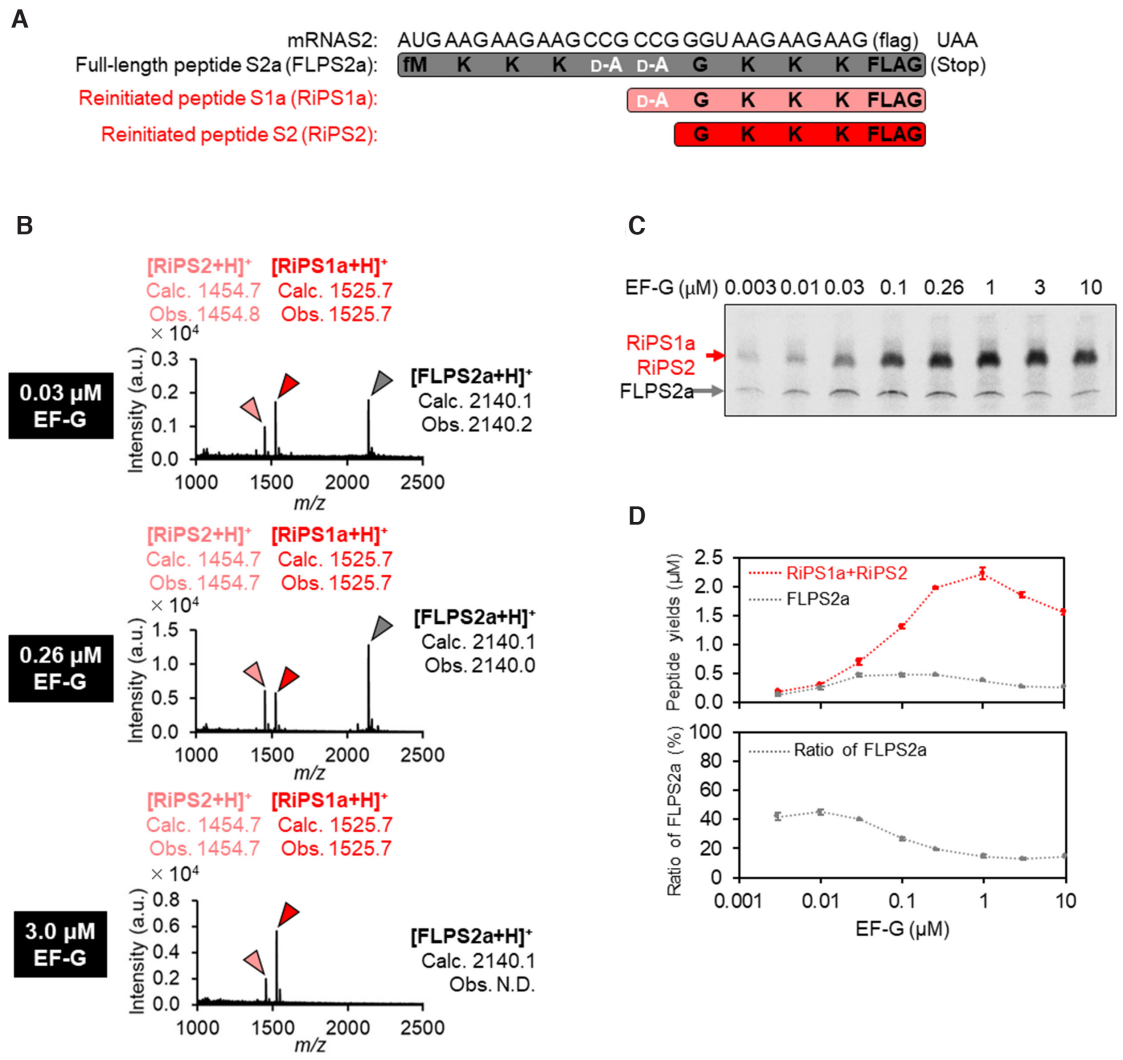
Mass spectrometric analysis and quantification of full-length and reinitiated peptides in incorporation of two consecutive d-alanines. (**A**) mRNA and expressed peptide sequences: full-length peptide (FLPS2a) and reinitiated peptides (RiPS1a and RiPS2). (**B**) Identification of translation products by MALDI-TOF MS. Peptides were expressed with 0.03, 0.26 or 3.0 μM EF-G. ‘Calc.’ and ‘Obs.’ indicate calculated and observed *m/z* values, respectively. (**C**) Titration of EF-G concentration in translation of mRNAS2. RiPS1a and RiPS2 could not be separated on the tricine-SDS-PAGE gel due to the only single amino acid difference between them. (**D**) Titration of EF-G concentration in translation of mRNAS2. Expressed peptides were radioisotope-labelled by [^14^C]-Asp in the C-terminal FLAG sequences, separated by tricine-SDS-PAGE, and quantified by autoradiography. Yields of peptides were quantified by autoradiography. *n* = 3.

Since not only Pro but also some kinds of nonproteinogenic amino acids, such as d-amino acids, cause inefficient peptidyl transfer reaction, we also evaluated the frequency of reinitiated peptide synthesis in two-consecutive incorporation of d-Ala into FLPS2a using mRNAS2 (Figure 3A). d-Ala was pre-charged onto an artificial suppressor tRNA, tRNA^GluE2^_CGG_, using flexizyme and introduced at CCG codons by means of genetic code reprogramming in a reconstituted translation system that lacks Pro. As a result, two types of reinitiated peptides, RiPS1a and RiPS2, were detected by MALDI-TOF MS (Figure 3B). RiPS1a and RiPS2 could not be separated on a tricine-SDS-PAGE gel due to the only single amino acid difference between them. Therefore, the sum of the expression level of these two peptides were estimated and compared to that of the full-length peptide, FLPS2a (Figure 3C, D). Consequently, the ratio of the RiPs significantly increased at higher EF-G concentrations. This tendency was consistent with that of consecutive Pro incorporation.

### Detection of drop-off peptides

In order to detect the drop-off peptide, translation of mRNA2 was conducted in the presence of [^14^C]-Lys so that all of FLP2, DoP2 and RiP2 were radio-labeled, and then PTH (15) was added after 20-min translation to remove tRNA moiety of the dropped peptidyl-tRNA (DoP2-tRNA). We observed a band corresponding to DoP2 at high PTH concentrations (Supplementary Figure S1B, DoP2), while the intensity of a band corresponding to DoP2-tRNA decreased by elevation of the PTH concentrations (Supplementary Figure S1B: DoP2-tRNA). However, the band of DoP2-tRNA did not completely disappear at 1 μM or above. We hypothesized that this is because unhydrolyzed [^14^C]-Lys-tRNA^Lys^ or peptidyl-tRNA protected from PTH by stalled ribosome overlapped on this band (29). In order to confirm this, not only PTH but also RNase A were added after 20-min translation, by which the PTH-sensitive band completely disappeared (Supplementary Figure S1C). This is probably because RNase A could decompose both dropped and ribosome-protected peptidyl-tRNAs. The band at the bottom that was observed in the presence of RNase A should be a peptidyl-adenosine (DoP2-adenosine) derived from the DoP2-tRNA (Supplementary Figure S1C: 50 μg/ml RNase A). Then, the DoP2 was also analyzed by LC-ESI MS (Figure 2D). Addition of RNase A gave two peaks bearing the same *m/z* values (Figure 2D, 2, *m/z* = 335.844, relative peak intensities were 671 and 3244, indicated by black arrow-heads), both of which were identified as DoP2-adenosine molecules by LC-ESI MS/MS analysis (Figure 2E). In the presence of PTH as well as RNase A, the intensity of DoP2-adenosine decreased by 10-fold, and instead a peak corresponding to DoP2 appeared (Figure 2D, 2 and 3: Relative peak intensity of DoP2-adenosine decreased from 671 to 76 at RT = 5.74 min, and from 3242 to 325 at RT = 6.15 min. The intensity of DoP2 was 2485.). The expression of DoP2-adenosine was significantly suppressed in the presence of EF-P (Figure 2D: 2 and 5, relative peak intensity change from 671 to 300 at RT = 5.74 min, and from 3244 to 1268 at RT = 6.15 min).

### Effects of length and sequence of nascent peptides on mistranslocation

**Figure 4.**
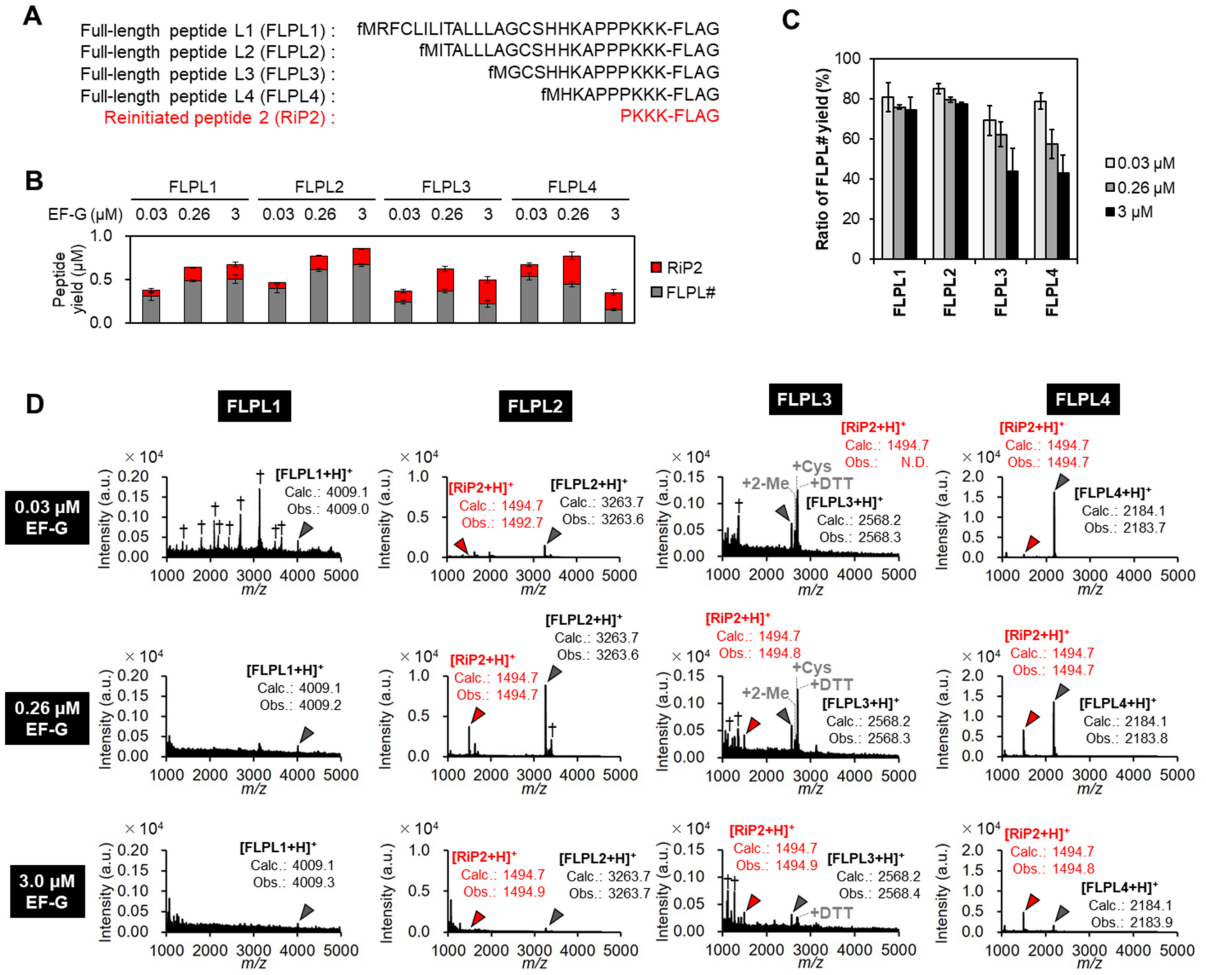
Effect of the length of nascent peptide chain on mistranslocation event. (**A**) Peptide sequences used in this analysis. The N-terminal region of NlpC protein containing a PPP motif was fused with KKK and FLAG peptide. FLPL1: The complete N-terminal peptide sequence derived from NlpC. FLPL2–4: Shortened variants of FLPL1. (**B**) Translation yields of FLPL1–4 and RiP2 in titration of EF-G in the absence of EF-P. Peptides were labeled by [^14^C]-Asp and quantified by autoradiography. *n* = 3. (**C**) Ratios of full-length peptides FLPL1–4. The ratios were calculated as (yield of FLPL#)/[(yield of FLPL#) + (yields of RiP2)] using the values obtained in (B). *n* = 3. (**D**) Identification of the translation products by MALDI-TOF MS. Peptides were expressed with 0.03, 0.26 or 3.0 μM EF-G in the absence of EF-P for 20 min. Grey arrowheads: FLPL1–4, red arrowheads: RiP2, *: Na^+^ or K^+^ adduct peaks, †: unidentified peaks. +2-ME, +Cys and + DTT are 2-mercaptoethanol, cysteine and dithiothreitol adducts, respectively. ‘Calc.’ and ‘Obs.’ indicate calculated and observed *m/z* values, respectively. N.D.: not detected.

As explained above, the length of nascent peptide of P-site peptidyl-tRNA should be one of critical determinants of drop-off frequency. Therefore, we examined different lengths of nascent peptides with EF-G titration (Figure 4A: FLPL1–4). Peptides were [^14^C]-Asp-labelled in the C-terminal FLAG, and analyzed by tricine-SDS-PAGE and autoradiography (Figure 4B, C). In the absence of EF-P, the expression of FLPL1–4 yielded the corresponding full-length peptides along with RiP2 (Figure 4B: FLPL1–4, grey bars; RiP2, red bars). At the high EF-G concentration (3 μM), shorter peptides, FLPL3 and 4, exhibited low FLPL ratios [FLPL3/(FLPL3 + RiP2) and FLPL4/(FLPL4 + RiP2), respectively], indicating more frequent peptidyl-tRNA drop-off due to their insufficient stabilization via interaction with the exit tunnel. On the other hand, longer peptides, FLPL1 and 2, exhibited significantly higher FLPL ratios. (Figure 4C: ratio of FLPL1, 2, 3 and 4 were 75, 78, 44 and 43% at 3 μM EF-G). The identities of FLPL1–4 and RiP2 were also confirmed by MALDI-TOF MS using non-radio-labeled peptides (Figure 4D).

**Figure 5.**
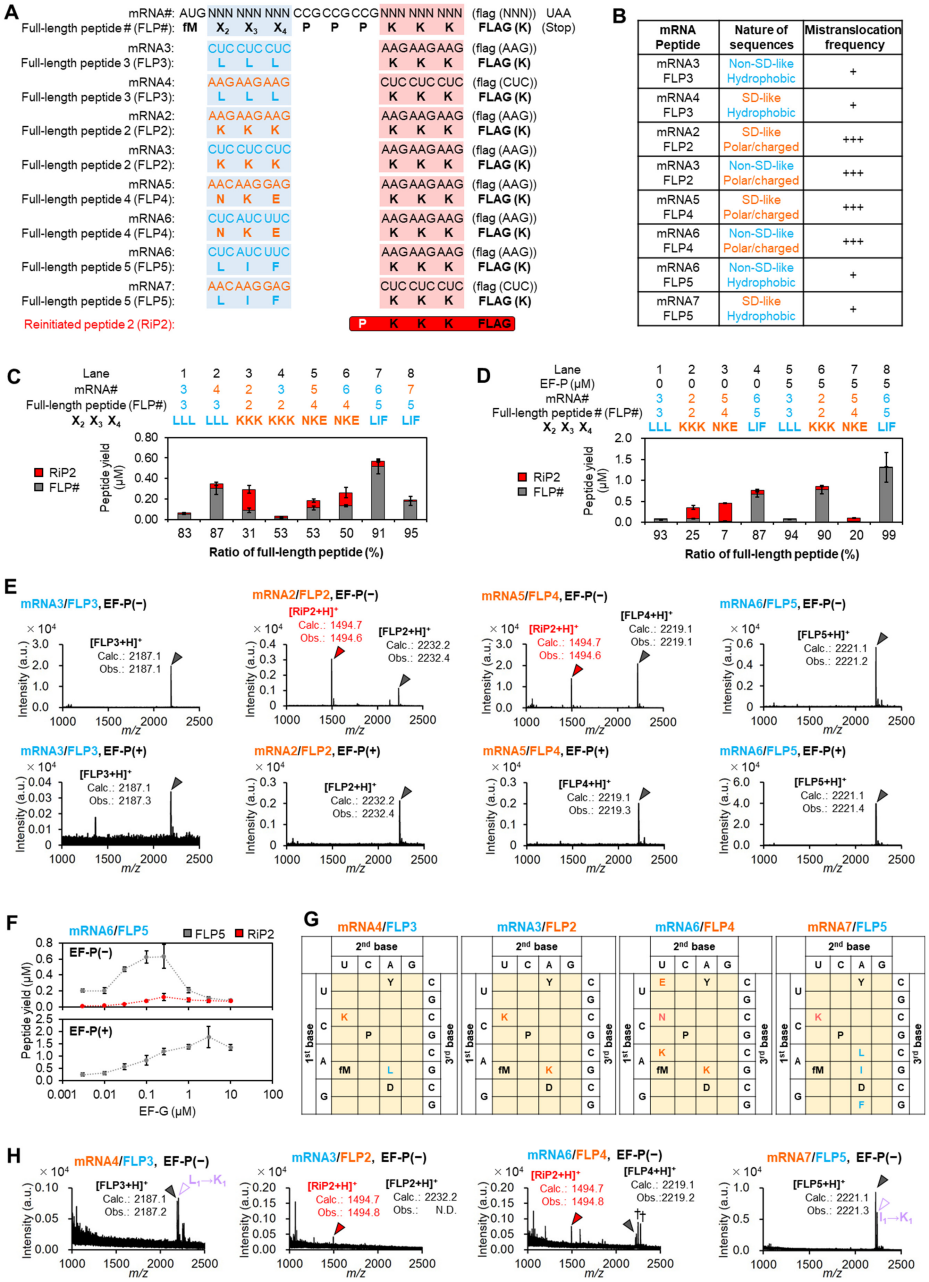
Drop-off-reinitiation caused by nascent peptide chain sequences. (**A**) Peptides (FLP2–5) and the corresponding mRNAs (mRNA2–7). The canonical genetic code assigns hydrophobic amino acids (sky-blue) to pyrimidine-rich codons (sky-blue), and polar amino acids (orange) to purine-rich codons (orange). Reprogrammed genetic codes (see also G) assign hydrophobic amino acids (sky-blue) to purine-rich codons (orange), and polar amino acids (orange) to pyrimidine-rich codons (sky-blue). The lysine residues that appear in the FLAG sequences are assigned to the codons indicated in parentheses. (**B**) Relationship between mistranslocation frequency and the nature of nascent peptide sequences/mRNA sequences. (**C**, **D**) Expression of the peptides (FLP2–5) from using the canonical or reprogrammed genetic codes in the absence (C: lanes 1–8, D: lanes 1–4) or presence of EF-P (D: lanes 5–8). *n* = 3. (**E**) Identification of the peptides expressed in (D). Grey arrowheads indicate peaks of FLP#s, and red arrowheads RiP2. ‘Calc.’ and ‘Obs.’ indicate calculated and observed *m/z* values, respectively. (**F**) Titration of EF-G in translation of mRNA6 to express FLP5 in the absence (top) and presence (bottom) of 5 μM EF-P. *n* = 3. (**G**) Reprogrammed codon tables used in translation of mRNA4 to FLP3, mRNA3 to FLP2, mRNA6 to FLP4 and mRNA7 to FLP5. (**H**) MALDI-TOF-MS identification of the peptides expressed in (C), lanes 2, 4, 6 and 8. Grey arrowheads indicate peaks of full-length peptides, and red ones peaks of reinitiated peptides. Purple open triangles indicate peaks corresponding to the FLP bearing L-to-K or I-to-K misincorporation. N.D.: not detected.

It has been also reported that certain nascent peptide sequences interact with the exit tunnel so strongly as to induce ribosomal stalling and slow down the peptidyl transfer (30). There is also an argument for a possibility that purine-rich Shine-Dalgarno (SD)-like mRNA ORF sequences interact with the pyrimidine-rich anti-SD sequence of 16S rRNA to cause ribosome stalling (31,32). Therefore, we next analyzed the effect of such nascent peptide and corresponding mRNA sequences. mRNA2 is translated into FLP2 containing positively-charged Lys-Lys-Lys residues prior to the Pro-Pro-Pro, which is encoded by a purine-rich SD-like sequence (AAGAAGAAG in Figure 5A). On the other hand, mRNA6 is translated into FLP5 bearing hydrophobic residues (Leu-Ile-Phe) encoded by non-SD-like pyrimidine-rich codons (CUCAUCUUC) followed by three consecutive Pro. Translation of mRNA6 into FLP5 yielded only small amount of RiP2 [Figure 5C, Supplementary Figure S1E: lane 7, Figure 5E: mRNA6/FLP5, EF-P(−)], whereas that of mRNA2 into FLP2 yielded a noticeable amount of RiP2 [Figure 5C, Supplementary Figure S1E: lane 3, Figures 1B, 5E: mRNA2/FLP2, EF-P(−)]. Another polar nascent peptide encoded by an SD-like sequence, AACAAGGAG/Asn-Lys-Glu (mRNA5/FLP4), also resulted in significantly higher amount of RiP2 synthesis compared with another hydrophobic nascent peptide encoded by a non-SD-like sequence, CUCCUCCUC/Leu-Leu-Leu (mRNA3/FLP3) [Figure 5C, Supplementary Figure S1E: lane 5 and 1, respectively, Figure 5E: mRNA5/FLP4 and mRNA3/FLP3, EF-P(−)]. We also confirmed that the ratio of reinitiated peptide increased at higher EF-G concentrations [Figure 5F, mRNA6/FLP5, EF-P(−)] and decreased in the presence of EF-P [Figure 5D, E, F: mRNA3/FLP3, mRNA2/FLP2, mRNA5/FLP4 and mRNA6/FLP5, EF-P(*+*)]. These results indicate that polar amino acids encoded by such SD-like mRNA sequences, mRNA2/FLP2 and mRNA5/FLP4, induce the mistranslocation event. However, it was yet unclear which is more or the most critical factor, mRNA context or peptide sequence. Therefore, we changed the relationship between codons and amino acids by means of genetic code reprograming (33) (Figure 5G), by which FLP3 bearing hydrophobic Leu-Leu-Leu was expressed from both the non-SD-like mRNA3 and SD-like mRNA4 (Figure 5C: lane 1 and 2, Figure 5E, H) and FLP5 with hydrophobic Leu-Ile-Phe was expressed from both the non-SD-like mRNA6 and SD-like mRNA7 (Figure 5C: lanes 7 and 8, Figure 5E, H). Ribosomal syntheses of FLP3 or FLP5 bearing the hydrophobic residues yielded the full-length peptide regardless of the mRNA context. Likewise, regardless of the context of mRNA template, ribosomal syntheses of FLP2 and FLP4 bearing the polar Lys-Lys-Lys and Asn-Lys-Glu residues, respectively, resulted in a noticeable amount of truncated RiP2 along with the full-length peptides (Figure 5C: lanes 3, 4, 5 and 6, Figure 5E, H). These results showed that induction of the drop-off-reinitiation event could be dictated by the peptide sequence rather than the mRNA context (Figure 5B). We propose a possibility that the electrostatic interaction between polar/charged nascent peptides and the exit tunnel may hold the peptidyl-tRNA at an inactive position in the peptidyl transferase center (PTC) and cause inefficient peptidyl transfer reaction.

### Comprehensive analysis of nascent peptide sequences that induce drop-off-reinitiation

**Figure 6.**
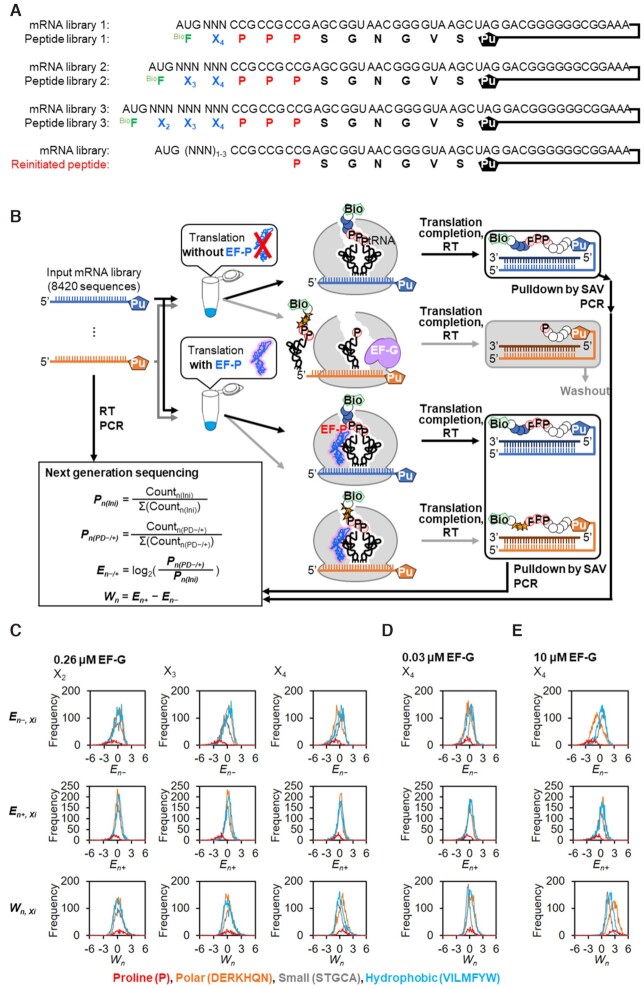
Comprehensive analysis of the effect of nascent peptide chain sequences. (**A**) mRNA and the corresponding peptide libraries. The peptide libraries have an N-terminal N-biotinyl-phenylalanine (^Bio^F, green), followed by 1–3 residues of any kinds of the 20 proteinogenic amino acids (X_2_, X_3_ and X_4_, blue), three consecutive prolines (P, red), and a linker peptide. The initiator AUG codon was reprogrammed to ^Bio^F, whereas the elongator AUG codon was assigned to Met. See also Supplementary Figure S3 for the details. UAG codon was made vacant to conjugate an mRNA and the corresponding peptide via Puromycin (Pu). (**B**) Schematic depiction of the comprehensive analysis of the effect of nascent peptide sequences on the three consecutive Pro incorporation. Puromycin-ligated mRNA libraries were translated with 0.03, 0.26 or 10 μM EF-G in the absence or presence of 5 μM EF-P, and then conjugated with the corresponding peptides via puromycin and reverse-transcribed. The resulting ^Bio^F-peptide-mRNA-cDNA conjugates were selectively pulled down by streptavidin magnetic beads. Truncated peptides caused by drop-off-reinitiation were not recovered due to the lack of N-terminal ^Bio^F. The pull-downed cDNA was amplified by PCR and sequenced. Proportion of a particular sequence ‘n’ in the initial library was estimated as *P_n(Ini)_*, and that in the pulled-down library obtained in the absence or presence of EF-P was estimated as *P_n(PD__−__)_* or *P_n(PD+)_*, respectively. Then, the enrichment of a sequence *n* (*E_n__−_* and *E_n+_*) were calculated by normalizing the *P_n(PD__−__)_* and *P_n(PD+)_* by *P_n(Ini)_*. The suppression of drop-off by EF-P was defined as *W_n_* = *E_n+_* − *E_n__−_*. RT: reverse transcription, SAV: streptavidin. (**C–E**) Histograms of *E_n__−_*, *E_n+_*, and *W_n_* sorted by the amino acid property of position X_2_, X_3_ and X_4_ with 0.26 μM (C), 0.03 μM (D), or 10 μM (E) EF-G. Histograms were separately drawn with red if X_i_ is proline (P), with orange if X_i_ is polar amino acid (D, E, R, K, H, Q, N), with grey if X_i_ is small amino acid (S, T, G, C, A), or with sky-blue if X_i_ is hydrophobic amino acid (V, I, L, M, F, Y, W).

To further explore nascent peptide sequences that induce drop-off-reinitiation, we applied a means of an mRNA display-based profiling (34). We constructed an mRNA library bearing 1–3 NNN random codons preceding three consecutive CCG Pro codons followed by a linker sequence (Figure 6A: a mixture of mRNA libraries 1, 2 and 3), where the N-terminal N-biotinyl-phenylalanine (^Bio^F) was installed by the initiation reprogramming. The peptide library expressed from this mRNA library was designed to contain 8,420 different peptide sequences to which 3′ end of the mRNA library was ligated via a puromycin linker for the formation of peptide-mRNA fusion (Figure 6B). We then translated the mRNA sequences with 0.03, 0.26 or 10 μM of EF-G in the absence or presence of EF-P, producing the respective mRNA-peptide fusions followed by reverse-transcription. Since only the full-length peptide-mRNA fusions should have the N-terminal ^Bio^F, they were selectively recovered by streptavidin beads via the N-terminal biotin. Thereby, the deep sequencing of such cDNAs would reveal the populations of fully expressed peptide sequences.

By processing the sequencing data, proportion of a particular peptide sequence ‘n’ in the initial library was estimated as *P_n__(Ini)_*, and that in the pulled-down library obtained in the absence or presence of EF-P was estimated as *P_n__(__P__D__−__)_* or *P_n__(__P__D__+__)_*, respectively. Then, enrichment of the peptide sequence n after pull-down compared to the initial library was evaluated as *E_n__−_* and *E_n__+_*, which were calculated by log_2_ [*P_n__(PD__−__)_* / *P_n__(Ini)_*] and log_2_ [*P_n__(PD+)_* / *P_n__(Ini)_*], respectively. These values reflect the efficiency of full-length peptide synthesis for the particular sequence n. The effect of EF-P on suppression of drop-off-reinitiation was evaluated as *W_n_* = *E_n+_* − *E_n__−_*. Then, *E_n__−_*, *E_n+_* and *W_n_* of all peptide sequences were summarized and visualized in heat maps (Supplementary Figure S4A–C). Those values were also plotted in histograms (Figure 6C–E, Supplementary Figure S5A, left), in which all peptides were categorized into four groups in terms of the property of amino acids at X_2_, X_3_ and X_4_ positions [polar residues (DERKHQN), small residues (STGCA), hydrophobic residues (VILMFYW) and Pro (P)].

The average *E_n_* values of peptides bearing Pro at X_2_, X_3_ and X_4_ were always less than zero regardless of EF-G concentration and presence of EF-P, indicating that Pro at these positions strongly promote drop-off-reinitiation (Figure 6C–E, S5A, left: *E_n-, X2__=__P_* = −1.11, −1.28, −1.37, *E_n__+__, X2__=__P_* = −0.81, −0.78, −1.31, *E_n__−__, X3__=__P_* = −1.77, −2.00, −2.23, *E_n__+__, X3__=__P_* = −1.08, −1.70, −2.16, *E_n__−__, X4__=__P_* = −0.74, −1.15, −1.95, *E_n__+__, X4__=__P_* = −0.61, −0.60, −1.10, for 0.03, 0.26 and 10 μM EF-G, respectively). Regarding other types of amino acids than Pro, no significant tendency in average *E_n_* values was observed at positions X_2_ and X_3_, whereas at position X_4_ polar residues showed significantly lower distribution in *E_n__−_*, indicating that drop-off-reinitiation is promoted by polar residues at position X_4_ in the absence of EF-P (Figure 6C–E: for instance, average *E_n__−_* = −0.70, 0.11 and −0.09 for polar, small and hydrophobic amino acids at 0.26 μM EF-G, average *E_n__−_* = −0.94, 0.30 and 0.10 at 10 μM EF-G). In addition, the difference of average *E_n_*_–_ between polar residues and others increased at higher EF-G concentration, showing that EF-G promotes drop-off-reinitiation (Figure 6C–E: Δ*E_n__−_* for small and hydrophobic residues were −0.83 and −0.40 at 0.03 μM EF-G, −0.81 and −0.61 at 0.26 μM EF-G, −1.24 and −1.04 at 10 μM EF-G). Since *E_n__+_* at position X_4_ did not show any polarity-dependent difference (Figure 6C–E), drop-off-reinitiation can be suppressed by EF-P, which resulted in higher *W_n_* values of polar residues.

In order to confirm the reliability of this profiling system, 6 representative sequences with the highest *E_n__−_* and 8 sequences with the lowest *E_n__−_* were individually translated *in vitro* at 10 μM EF-G in the absence or presence of EF-P, and quantified by LC-ESI MS (Supplementary Figure S5B, indicated by black arrows, S5E). The sequences with high *E_n__−_* could be efficiently translated into full-length peptides along with only a trace amount of reinitiated peptide RiPX even in the absence of EF-P (Supplementary Figure S5F, blue sequences, S5B). On the other hand, the sequences with low *E_n__−_* could not be translated into full-length at all in the absence of EF-P (Supplementary Figure S5F, orange sequences), but EF-P enabled expression of full-length peptides (Supplementary Figure S5G). We also performed sequence profiling by replacing the triple Pro of the mRNA library with a triple Gly, in which *E_n__−_* and *E_n+_* were less biased than those of the triple-Pro library, and EF-P had no effect as expected (Supplementary Figure S5A, right, S5D, S4D–F).

### In-cell reinitiated peptide synthesis caused by drop-off-reinitiation

Our *in vitro* studies described above undoubtedly have shown that the occurrence of drop-off-reinitiation led to an accumulation of the reinitiated peptide during the translation, so we wondered if this could be observed even in a cellular system. For this study, we chose five *Escherichia coli* proteins, YjgZ, PrpR, YhhM, RutD and YdcO, which have two or three consecutive Pro residues at the N-terminal region. We first set up *in vitro* translation experiments where the respective peptides corresponding to the N-terminal region (14–19 amino acid residues) of these proteins and observed their expression in the absence and presence of EF-P and PTH (Supplementary Figure S6A, FLPN1–5). The respective peptides were radio-labelled with [^35^S]-methionine (Met or M) at the N-terminus and analyzed by tricine-SDS-PAGE and autoradiography. The bands whose intensities increased in the absence of EF-P and PTH were derived from the dropped peptidyl-tRNA (Supplementary Figure S6B, green arrows). In order to confirm the identities of both DoP and RiP, non-labelled peptides were also expressed in the absence and presence of EF-P (both in the presence of PTH) and subject to LC-ESI MS analysis (Supplementary Figure S6C–G). We observed DoPs originating from peptidyl-tRNA drop-off and RiPs originating from reinitiation at various positions. Notably, expression of FLPN1 (YjgZ), FLPN2 (PrpR) and FLPN4 (RutD) resulted in mainly full-length peptides and relatively small amounts of truncated peptides, whereas FLPN3 (YhhM) and FLPN5 (YdcO) showed severe peptidyl-tRNA drop-off and reinitiation, leading to accumulation of more DoPs and RiPs. Interestingly, in the expression of FLPN5 (MRLFSIPPPTLLAGFLAVL-FLAG), MRLFSIP was the dominant DoP, indicating that peptidyl-tRNA drop-off mainly occurred at the first Pro residue; However, not PPTLLAGFLAVL-FLAG but PTLLAGFLAVL-FLAG and TLLAGFLAVL-FLAG were the dominant RiPs, indicating that the translation reinitiated from the third Pro or the next Thr by skipping one or two Pro residues. This is probably because the drop-off event occurred successively at the Pro-Pro-Pro sequence.

**Figure 7.**
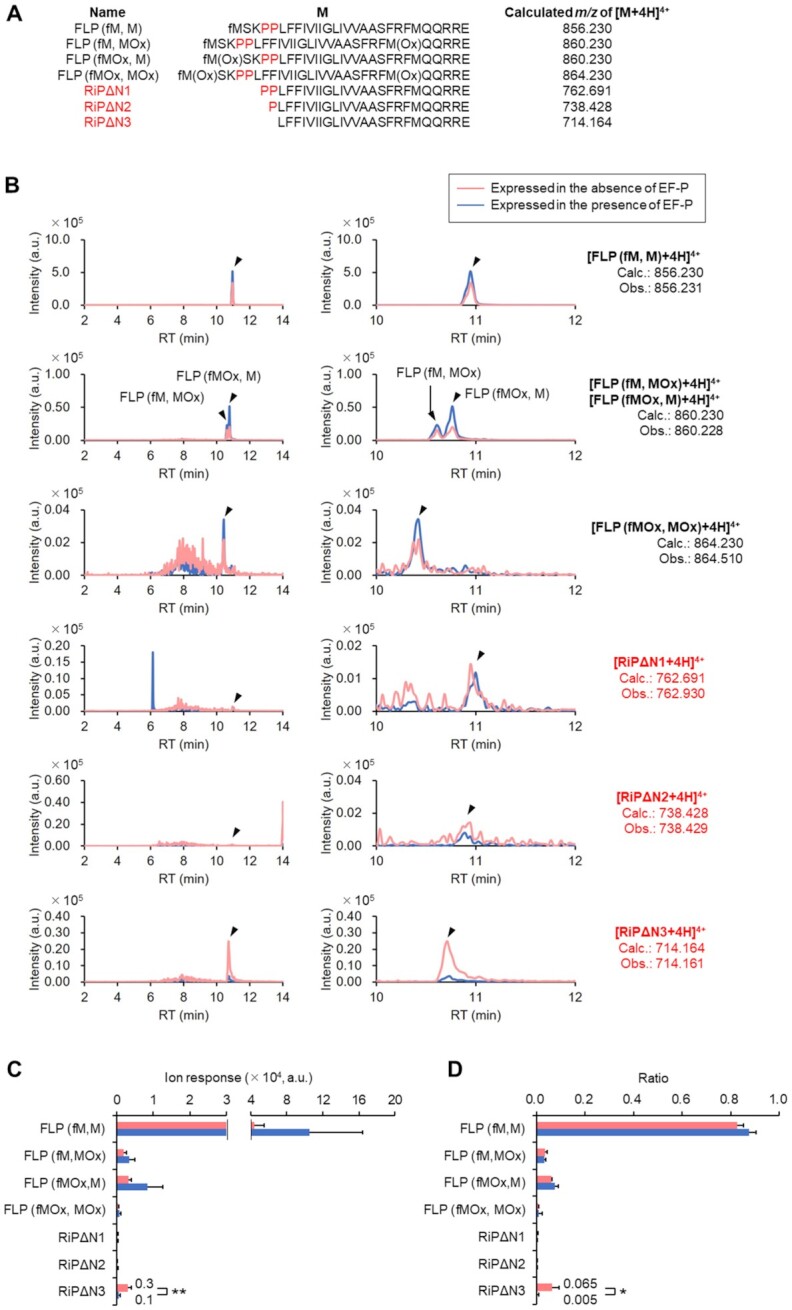
LC-ESI MS analysis of N-terminal fragments of YhhM-fh expressed in *E. coli*. (**A**) N-terminal peptide sequences generated by Lys-N digestion of full-length YhhM-fh (FLP) and reinitiated YhhM-fh lacking its N-terminal amino acids (RiPΔN1, RiPΔN2 and RiPΔN3). MOx: Methionine oxidized on its sidechain thioether. (**B**) Full-range (left) and magnified (right) XICs of N-terminal peptides generated by Lys-N digestion of YhhM-fh expressed alone (red) and co-expressed with EF-P (blue). XICs were extracted on the observed *m*/*z* values (Obs.). (**C**, **D**) LC-ESI MS quantification of ion response (C) and ratio (D) of N-terminal fragments generated by Lys-N digestion of YhhM-fh expressed in the absence or presence of EF-P. Ratios were calculated as (ion response of an N-terminal fragment)/(sum of ion responses of all the N-terminal fragments). SD of three independent experiments using YhhM-fh expressed without EF-P and SD of four independent experiments using YhhM-fh expressed with EF-P are shown. *P < 0.1 in Welch's *t* test. **P < 0.05 in Welch's *t* test.

To this end, we chose YhhM as a model and conducted its cellular expression where FLAG and 6 × His peptide motifs were added to the C-terminus, referred to as YhhM-fh in order to facilitate the isolation of the full-length as well as reinitiated proteins. This YhhM-fh sequence was cloned into pBAD-HisA vector under the regulation of arabinose promoter. YhhM-fh was expressed in either EF-P-knockout *E. coli* (*E. coli* Δ*efp*) (35,36) or EF-P-expressing *E. coli* bearing modified pBAD vector that complements *efp* gene (Supplementary Figure S7A, *E. coli* Δ*efp/+efp*). In *E. coli* Δ*efp/+efp*, we confirmed ϵ(*R*)-β-lysyl-hydroxylation on Lys34 of EF-P, which is introduced by endogenous EpmA/B/C enzymes (37–39) (Supplementary Figure S7B, C). Affinity-purified YhhM-fh was digested by Lys-N (40) and analyzed by LC-ESI MS/MS, by which N-terminal fragments derived from the full-length YhhM-fh and those lacking 3–5 N-terminal amino acids were detected (Figure 7A, B, Supplementary Figure S6H–U, S8). Note that Lys3 was not cleaved by Lys-N (Figure 7A, B, Supplementary Figure S6H–K, O–R), probably because the N-terminal fMet-Ser prior to Lys3 was too short to be recognized by Lys-N. While the intensity of the Lys-N-digested fragment derived from the full-length YhhM-fh [FLP (fM, M)] was comparable between Δ*efp* and Δ*efp/+efp* (Figure 7C, the relative intensities were 4.9 ± 1.1 × 10^4^ and 11 ± 6 × 10^4^, respectively; Figure 7D, their fractions were 93 ± 3% and 99 ± 4%, respectively), the intensity of the RiP fragment lacking fMet-Ser-Lys-Pro-Pro (RiPΔN3) was higher in Δ*efp* than in Δ*efp/+efp* (Figure 7C, their relative intensities were 3.1 ± 0.9 × 10^3^ and 6 ± 4 × 10^2^, respectively, with *P* < 0.05 in Welch's t test; Figure 7D, their fractions were 6 ± 3% and 0.5 ± 0.3%, respectively, with *P* < 0.1 in Welch's *t* test). This result suggests that the drop-off-reinitiation event occurred more frequently in the absence of EF-P than its presence because of inefficient peptidyl transfer. Among a series of RiP fragments lacking several N-terminal amino acids, RiPΔN3 was clearly dominant, indicating that the peptidyl transfer between peptidyl-diprolyl-tRNA and leucyl-tRNA was inefficient in the expression of YhhM-fh in the absence of EF-P (Figure 7C, the relative intensity of RiPΔN1, 2 and 3 expressed in the absence of EF-P was 1.6 ± 1.2 × 10^2^, 0.8 ± 0.3 × 10^2^ and 3.1 ± 0.9 × 10^3^, respectively, while those in the presence of EF-P was 1.5 ± 1.5 × 10^2^, 1.4 ± 0.9 × 10^2^ and 6 ± 4 × 10^2^, respectively; Figure 7D, the fractions of RiPΔN1, 2 and 3 expressed in the absence of EF-P were 0.3 ± 0.2%, 0.15 ± 0.05% and 6 ± 3%, respectively, while those in the presence of EF-P was 0.2 ± 0.1%, 0.12 ± 0.07% and 0.5 ± 0.3%, respectively).

In this cell-based assay, an overexpressed protein, YhhM-fh, was analyzed because the use of a specific tag sequence was necessary for the efficient isolation and detection of the fragments. However, the result has strongly supported a possibility where the drop-off-reinitiation could occur in the cellular system. The evolutionary importance of EF-P is also supported by this result, in turn suggesting why EF-P and its homologs are widely conserved in the three domains of life. Thus, the drop-off-reinitiation can frequently occur without EF-P, resulting in an accumulation of aberrant proteins that lack their N-terminal region.

## DISCUSSION

**Figure 8.**
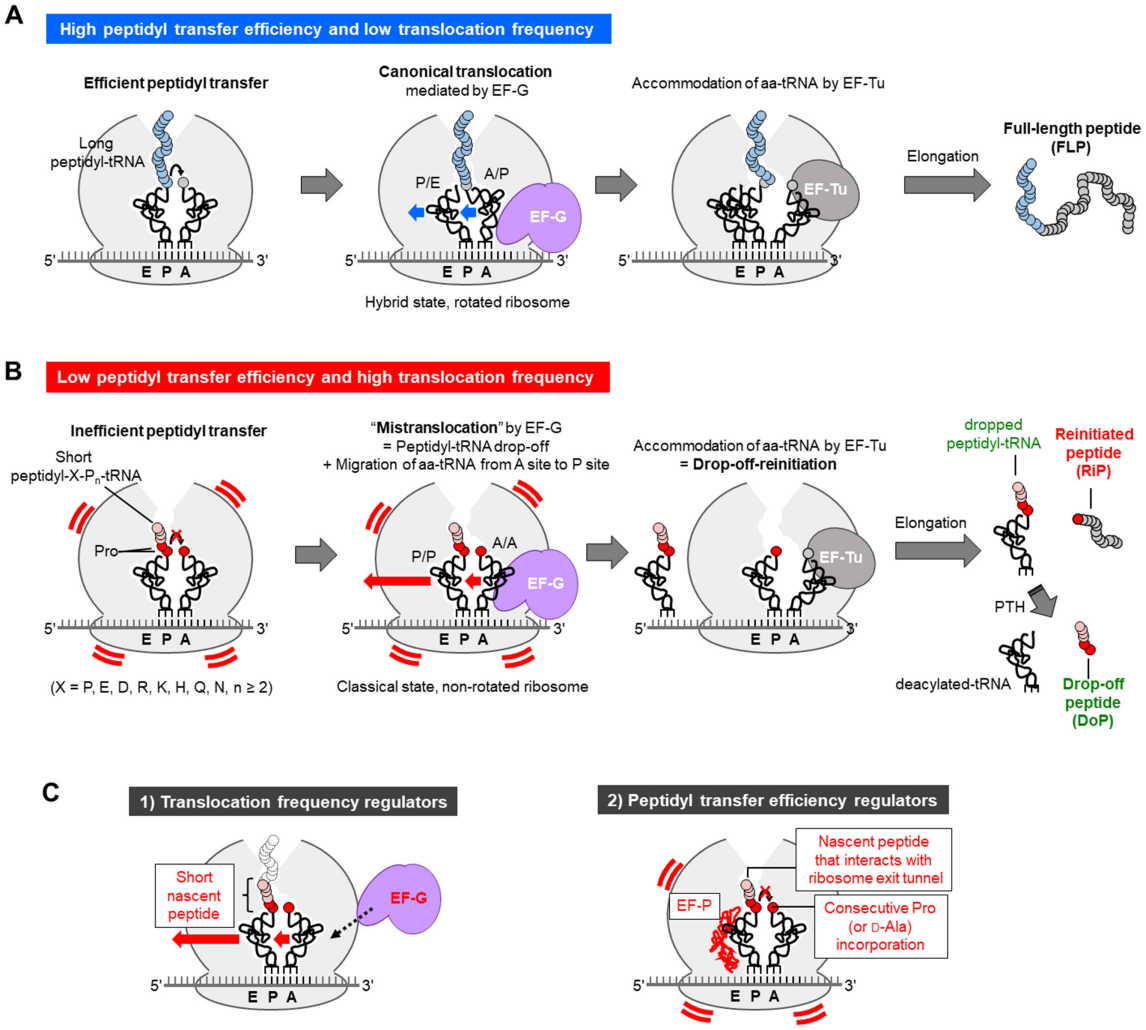
Overview of EF-G-driven mistranslocation and drop-off-reinitiation. (**A**) Schematic illustration of peptide elongation in the canonical translation. Ribosome catalyzes peptidyl transfer by which a new peptide bond is formed between a nascent peptide on P-site peptidyl-tRNA (blue) and an amino acid on A-site aminoacyl-tRNA (grey). In the canonical translocation mediated by EF-G (purple), P-site deacylated-tRNA and A-site peptidyl-tRNA migrate to E site and P site, respectively. In accommodation, an aminoacyl-tRNA corresponding to the next A-site codon is delivered by EF-Tu. By repeating the three reactions, a full-length peptide is synthesized. (**B**) Schematic illustration of P-site peptidyl-tRNA drop-off and generation of reinitiated peptide caused by mistranslocation and drop-off-reinitiation. Consecutive incorporation of prolines (P, red) makes peptidyl transfer reaction inefficient. Before completion of the inefficient peptidyl transfer, drop-off of the peptidyl-tRNA and migration of A-site aminoacyl-tRNA to P site occur, which we refer to as mistranslocation. Then, translation is reinitiated by accommodation of a new aminoacyl-tRNA on to A site to give a reinitiated peptide, RiP. (**C**) Candidates of translocation frequency regulators (1, left) and peptidyl transfer frequency regulators (2, right). Those indicated by red letters are confirmed translocation frequency regulators (short nascent peptide and EF-G) and peptidyl transfer frequency regulators (consecutive Pro incorporation, EF-P and sequence of nascent peptide).

Although the mechanism and processing pathway of peptidyl-tRNA drop-off event have been previously studied (2–4), the fate of the ribosome complex that lost the peptidyl-tRNA has not been well understood. Here, we reported for the first time that such a ribosome complex is subject to EF-G-dependent translocation and then reinitiation occurs to yield an RiP (Figure 8B). We referred this event to as drop-off-reinitiation and confirmed that it occurs both *in vitro* and in cells. The frequency of drop-off-reinitiation is regulated by the following two factors: (i) translocation frequency and (ii) peptidyl transfer efficiency (Figure 8C).

EF-G is the critical regulator that determines translocation frequency (Figure 8C left). In the canonical translocation, EF-G mediates migration of P/E-site deacylated-tRNA and A/P-site peptidyl-tRNA in the hybrid state to E/E and P/P site, respectively, after completion of peptidyl transfer (Figure 8A) (17). In contrast, in the mistranslocation, P/P-site peptidyl-tRNA and A/A-site aminoacyl-tRNA migrate toward E/E and P/P site, respectively, before peptidyl transfer completes (Figure 8B). It has been previously reported that N-acetyl-Phe-tRNA in the P site can translocate toward E site, albeit less efficiently than the canonical translocation (41). Presumably, the nascent peptide on the peptidyl-tRNA perturbs the interaction between the tRNA and 23S rRNA in the E site and thereby makes the translocation less efficient (42,43). It has been also reported that a replacement of the peptidyl group of A-site tRNA with an aminoacyl group inhibits the translocation to P site (44). Moreover, since EF-G should bind preferably to the rotated ribosome/tRNAs in the hybrid state compared to those in the non-rotated classical state, the mistranslocation would not efficiently take place at a low concentration of EF-G. Therefore, an elevation of the EF-G concentration should be required to induce the mistranslocation with a higher frequency, yielding more RiPs. The concentration of EF-G in *E. coli* cell is estimated to be approximately twofold higher than that of 70S ribosome (45), which corresponds to 2.4 μM in the case of our *in vitro* translation system whose concentration of ribosome is 1.2 μM. In this EF-G concentration range, peptidyl-tRNA drop-off is induced but suppressed by EF-P *in vitro* (Figure 1B). A similar trend was observed in the in-cell experiment, in which the YhhM-fh lacking its N-terminus was detected but largely suppressed by EF-P.

Susceptibility of P-site peptidyl-tRNA to drop-off is another regulator of the drop-off-reinitiation frequency (Figure 8C left). Shorter nascent peptides of peptidyl-tRNA increased the drop-off susceptibility due to the insufficient interaction with the exit tunnel, and resulted in more RiP synthesis (Figure 4). In *E. coli* cells, there are 2115 proteins and putative proteins containing two or more consecutive Pro, 6% of which are located within N-terminal 24 amino acid residues (46). When the nascent peptide on peptidyl-Pro-tRNA is shorter than the depth of the exit tunnel, RiP synthesis possibly tends to occur via drop-off-reinitiation. If that is the case, the population of RiPs should not be ignorable, although more comprehensive in-cell studies should be performed to verify this.

As for the regulators of peptidyl transfer efficiency (Figure 8C right), we evaluated the effect of consecutive Pro residues in model peptides and proteins. Increasing the number of consecutive Pro, RiPs derived from mistranslocation were more frequently observed. The inefficient peptidyl transfer observed for the consecutive Pro elongation can be alleviated by adding EF-P to the translation system, by which drop-off-reinitiation was also suppressed. EF-P recognizes the P-site peptidyl-Pro-tRNA^Pro^ and stabilizes its CCA end for shaping suitable geometry for peptidyl transfer (47). In addition to consecutive Pro, certain nascent peptide sequences that interact with the ribosomal exit tunnel also make peptidyl transfer inefficient. We conducted a comprehensive analysis of 8,420 nascent peptide sequences preceding the three consecutive Pro, showing that polar amino acids adjacent to the Pro stretch significantly promoted drop-off-reinitiation, which was also suppressed by EF-P (Figure 6, Supplementary Figure S4A–C). Our result looks consistent with previous related studies where Pro and polar amino acids, such as Arg, Asp, His and Lys, were preferred at position X of the stalling motif, XPP or XPPP, although the contribution of polar residues was not clearly discussed in the papers (48–50). These studies also showed that the sequence dependent ribosome stalling was alleviated by EF-P. Not only consecutive Pro motifs but also other types of arrest peptides and ribosome destabilizing peptides have also been reported. For example, expression of TnaC stalls with the P-site peptidyl-Pro-tRNA (51), whereas that of SecM stalls with the A-site Pro-tRNA (52). MgtL peptide (MEPDPTPLPRRRLKLFR) and peptides bearing 10 consecutive acidic residues also destabilize ribosome upon Mg^2+^ starvation, leading to a dissociation of the ribosomal subunits and eventually translation abortion (53). Although the mechanisms for the ribosomal stalling and destabilization are not fully understood, both Pro and polar/acidic residues in such peptides may force the peptidyl-tRNA inactive for peptidyl transfer reaction by electrostatic repulsion with the tunnel. Therefore, these peptide sequences also act as potent drop-off-reinitiation inducers (Figure 8C right).

Drop-off-reinitiation induced by mistranslocation is distinct from other ribosome rescue pathways mediated by specific quality control factors, such as tmRNA/SmpB (54,55), ArfA (56) and ArfB (57). These rescue pathways do not generate RiPs from the stalled ribosome. Since both A and P sites are occupied by peptidyl- and aminoacyl-tRNAs, respectively, in the case of drop-off-reinitiation, these rescue factors cannot enter the A site where they work. Although we could detect RiPs in *E. coli*, it is still unclear whether such truncated peptides/proteins are rapidly and selectively degraded or can circumvent such degradation pathway to survive. If the N-end rule pathway applies to the truncated proteins (58), the lack of fMet and the alternate N-terminal amino acids (typically Pro) would play a role in protein stability. Elucidation of these issues should be important for future works.

## Supplementary Material

gkac068_Supplemental_Files

## References

[1] Schmeing T.M. , Ramakrishnan V. What recent ribosome structures have revealed about the mechanism of translation. Nature. 2009; 461:1234–1242. 19838167 10.1038/nature08403

[2] Menninger J.R. Peptidyl transfer RNA dissociates during protein synthesis from ribosomes of *Escherichia**coli*. J. Biol. Chem. 1976; 251:3392–3398. 776968

[3] Cruz-Vera L.R. , Magos-Castro M.A., Zamora-Romo E., Guarneros G. Ribosome stalling and peptidyl-tRNA drop-off during translational delay at AGA codons. Nucleic Acids Res. 2004; 32:4462–4468. 15317870 10.1093/nar/gkh784 PMC516057

[4] de Valdivia E.I.G. , Isaksson L.A. Abortive translation caused by peptidyl-tRNA drop-off at NGG codons in the early coding region of mRNA. FEBS J. 2005; 272:5306–5316. 16218960 10.1111/j.1742-4658.2005.04926.x

[5] Fujino T. , Goto Y., Suga H., Murakami H. Reevaluation of the D-amino acid compatibility with the elongation event in translation. J. Am. Chem. Soc. 2013; 135:1830–1837. 23301668 10.1021/ja309570x

[6] Muto H. , Ito K. Peptidyl-prolyl-tRNA at the ribosomal P-site reacts poorly with puromycin. Biochem. Biophys. Res. Commun. 2008; 366:1043–1047. 18155161 10.1016/j.bbrc.2007.12.072

[7] Doerfel L.K. , Wohlgemuth I., Kothe C., Peske F., Urlaub H., Rodnina M.V. EF-P is essential for rapid synthesis of proteins containing consecutive proline residues. Science. 2013; 339:85–88. 23239624 10.1126/science.1229017

[8] Ude S. , Lassak J., Starosta A.L., Kraxenberger T., Wilson D.N., Jung K. Translation elongation factor EF-P alleviates ribosome stalling at polyproline stretches. Science. 2013; 339:82–85. 23239623 10.1126/science.1228985

[9] Pavlov M.Y. , Watts R.E., Tan Z., Cornish V.W., Ehrenberg M., Forster A.C. Slow peptide bond formation by proline and other N-alkylamino acids in translation. Proc. Natl. Acad. Sci. U.S.A. 2009; 106:50–54. 19104062 10.1073/pnas.0809211106 PMC2629218

[10] Otaka T. , Kaji A. Release of (oligo) peptidyl-tRNA from ribosomes by erythromycin A. Proc. Natl. Acad. Sci. U.S.A. 1975; 72:2649–2652. 1101261 10.1073/pnas.72.7.2649 PMC432827

[11] Menninger J.R. , Otto D.P. Erythromycin, carbomycin, and spiramycin inhibit protein synthesis by stimulating the dissociation of peptidyl-tRNA from ribosomes. Antimicrob. Agents Chemother. 1982; 21:811–818. 6179465 10.1128/aac.21.5.811 PMC182017

[12] Lovmar M. , Tenson T., Ehrenberg M. Kinetics of macrolide action. J. Biol. Chem. 2004; 279:53506–53515. 15385552 10.1074/jbc.M401625200

[13] Tenson T. , Lovmar M., Ehrenberg M. The mechanism of action of macrolides, lincosamides and streptogramin B reveals the nascent peptide exit path in the ribosome. J. Mol. Biol. 2003; 330:1005–1014. 12860123 10.1016/s0022-2836(03)00662-4

[14] Mankin A.S. Macrolide myths. Curr. Opin. Microbiol. 2008; 11:414–421. 18804176 10.1016/j.mib.2008.08.003 PMC3874820

[15] Das G. , Varshney U. Peptidyl-tRNA hydrolase and its critical role in protein biosynthesis. Microbiology. 2006; 152:2191–2195. 16849786 10.1099/mic.0.29024-0

[16] Kang T.J. , Suga H. Translation of a histone H3 tail as a model system for studying peptidyl-tRNA drop-off. FEBS Lett. 2011; 585:2269–2274. 21627973 10.1016/j.febslet.2011.05.051

[17] Rodnina M.V. , Savelsbergh A., Katunin V.I., Wintermeyer W. Hydrolysis of GTP by elongation factor G drives tRNA movement on the ribosome. Nature. 1997; 385:37–41. 8985244 10.1038/385037a0

[18] Gao Y.G. , Selmer M., Dunham C.M., Weixlbaumer A., Kelley A.C., Ramakrishnan V. The structure of the ribosome with elongation factor G trapped in the posttranslocational state. Science. 2009; 326:694–699. 19833919 10.1126/science.1179709 PMC3763468

[19] Saito H. , Kourouklis D., Suga H. An *in vitro* evolved precursor tRNA with aminoacylation activity. EMBO J. 2001; 20:1797–1806. 11285242 10.1093/emboj/20.7.1797 PMC145511

[20] Murakami H. , Ohta A., Ashigai H., Suga H. A highly flexible tRNA acylation method for non-natural polypeptide synthesis. Nat. Methods. 2006; 3:357–359. 16628205 10.1038/nmeth877

[21] Johansson M. , Ieong K.W., Trobro S., Strazewski P., Åqvist J., Pavlov M.Y., Ehrenberg M. pH-sensitivity of the ribosomal peptidyl transfer reaction dependent on the identity of the A-site aminoacyl-tRNA. Proc. Natl. Acad. Sci. U.S.A. 2011; 108:79–84. 21169502 10.1073/pnas.1012612107 PMC3017169

[22] Tanner D.R. , Cariello D.A., Woolstenhulme C.J., Broadbent M.A., Buskirk A.R. Genetic identification of nascent peptides that induce ribosome stalling. J. Biol. Chem. 2009; 284:34809–34818. 19840930 10.1074/jbc.M109.039040 PMC2787343

[23] Heurgué-Hamard V. , Karimi R., Mora L., MacDougall J., Leboeuf C., Grentzmann G., Ehrenberg M., Buckingham R.H. Ribosome release factor RF4 and termination factor RF3 are involved in dissociation of peptidyl-tRNA from the ribosome. EMBO J. 1998; 17:808–816. 9451005 10.1093/emboj/17.3.808 PMC1170429

[24] Gong M. , Cruz-Vera L.R., Yanofsky C. Ribosome recycling factor and release factor 3 action promotes TnaC-peptidyl-tRNA dropoff and relieves ribosome stalling during tryptophan induction of tna operon expression in *Escherichia**coli*. J. Bacteriol. 2007; 189:3147–3155. 17293419 10.1128/JB.01868-06 PMC1855834

[25] Singh N.S. , Ahmad R., Sangeetha R., Varshney U. Recycling of ribosomal complexes stalled at the step of elongation in *Escherichia coli*. J. Mol. Biol. 2008; 380:451–464. 18565340 10.1016/j.jmb.2008.05.033

[26] Katoh T. , Wohlgemuth I., Nagano M., Rodnina M.V., Suga H. Essential structural elements in tRNA^Pro^ for EF-P-mediated alleviation of translation stalling. Nat. Commun. 2016; 7:11657. 27216360 10.1038/ncomms11657 PMC4890201

[27] Alejo J.L. , Blanchard S.C. Miscoding-induced stalling of substrate translocation on the bacterial ribosome. Proc. Natl. Acad. Sci. U.S.A. 2017; 114:E8603–E8610. 28973849 10.1073/pnas.1707539114 PMC5642701

[28] Gamper H.B. , Masuda I., Frenkel-Morgenstern M., Hou Y.M. Maintenance of protein synthesis reading frame by EF-P and m^1^G37-tRNA. Nat. Commun. 2015; 6:7226. 26009254 10.1038/ncomms8226 PMC4445466

[29] Vogel Z. , Vogel T., Zamir A., Elson D The protection by 70 S ribosomes of N-acyl-aminoacyl-tRNA against cleavage by peptidyl-tRNA hydrolase and its use to assay ribosomal association. Eur. J. Biochem. 1971; 21:582–592. 4938621 10.1111/j.1432-1033.1971.tb01504.x

[30] Ito K. , Chiba S. Arrest peptides: cis-acting modulators of translation. Annu. Rev. Biochem. 2013; 82:171–202. 23746254 10.1146/annurev-biochem-080211-105026

[31] Li G.W. , Oh E., Weissman J.S. The anti-Shine-Dalgarno sequence drives translational pausing and codon choice in bacteria. Nature. 2012; 484:538–541. 22456704 10.1038/nature10965 PMC3338875

[32] Mohammad F. , Woolstenhulme C.J., Green R., Buskirk A.R. Clarifying the translational pausing landscape in bacteria by ribosome profiling. Cell Rep. 2016; 14:686–694. 26776510 10.1016/j.celrep.2015.12.073 PMC4835026

[33] Goto Y. , Katoh T., Suga H. Flexizymes for genetic code reprogramming. Nat. Protoc. 2011; 6:779–790. 21637198 10.1038/nprot.2011.331

[34] Rogers J.M. , Passioura T., Suga H. Nonproteinogenic deep mutational scanning of linear and cyclic peptides. Proc. Natl. Acad. Sci. U.S.A. 2018; 115:10959–10964. 30301798 10.1073/pnas.1809901115 PMC6205457

[35] Baba T. , Ara T., Hasegawa M., Takai Y., Okumura Y., Baba M., Datsenko K.A., Tomita M., Wanner B.L., Mori H. Construction of *Escherichia**coli* K-12 in-frame, single-gene knockout mutants: the Keio collection. Mol. Syst. Biol. 2006; 2:2006.0008. 10.1038/msb4100050 PMC1681482 16738554

[36] Yamamoto N. , Nakahigashi K., Nakamichi T., Yoshino M., Takai Y., Touda Y., Furubayashi A., Kinjyo S., Dose H., Hasegawa M. et al. Update on the Keio collection of *Escherichia**coli* single-gene deletion mutants. Mol. Syst. Biol. 2009; 5:335. 20029369 10.1038/msb.2009.92 PMC2824493

[37] Roy H. , Zou S.B., Bullwinkle T.J., Wolfe B.S., Gilreath M.S., Forsyth C.J., Navarre W.W., Ibba M. The tRNA synthetase paralog PoxA modifies elongation factor-P with (R)-β-lysine. Nat. Chem. Biol. 2011; 7:667–669. 21841797 10.1038/nchembio.632 PMC3177975

[38] Yanagisawa T. , Sumida T., Ishii R., Takemoto C., Yokoyama S. A paralog of lysyl-tRNA synthetase aminoacylates a conserved lysine residue in translation elongation factor P. Nat. Struct. Mol. Biol. 2010; 17:1136–1143. 20729861 10.1038/nsmb.1889

[39] Peil L. , Starosta A.L., Virumäe K., Atkinson G.C., Tenson T., Remme J., Wilson D.N. Lys34 of translation elongation factor EF-P is hydroxylated by YfcM. Nat. Chem. Biol. 2012; 8:695–697. 22706199 10.1038/nchembio.1001

[40] Nonaka T. , Hashimoto Y., Takio K. Kinetic characterization of lysine-specific metalloendopeptidases from *Grifola**frondosa* and *Pleurotus**ostreatus* fruiting bodies. J. Biochem. 1998; 124:157–162. 9644258 10.1093/oxfordjournals.jbchem.a022074

[41] Fredrick K. , Noller H.F. Accurate translocation of mRNA by the ribosome requires a peptidyl group or its analog on the tRNA moving into the 30S P site. Mol. Cell. 2002; 9:1125–1131. 12049747 10.1016/s1097-2765(02)00523-3

[42] Lill R. , Lepier A., Schwägele F., Sprinzl M., Vogt H., Wintermeyer W. Specific recognition of the 3′-terminal adenosine of tRNA^Phe^ in the exit site of *Escherichia coli* ribosomes. J. Mol. Biol. 1988; 203:699–705. 2463367 10.1016/0022-2836(88)90203-3

[43] Lill R. , Robertson J.M., Wintermeyer W. Binding of the 3′ terminus of tRNA to 23S rRNA in the ribosomal exit site actively promotes translocation. EMBO J. 1989; 8:3933–3938. 2583120 10.1002/j.1460-2075.1989.tb08574.x PMC402085

[44] Walker S.E. , Shoji S., Pan D., Cooperman B.S., Fredrick K. Role of hybrid tRNA-binding states in ribosomal translocation. Proc. Natl. Acad. Sci. U.S.A. 2008; 105:9192–9197. 10.1073/pnas.0710146105 PMC2453706 18591673

[45] Schmidt A. , Kochanowski K., Vedelaar S., Ahrné E., Volkmer B., Callipo L., Knoops K., Bauer M., Aebersold R., Heinemann M. The quantitative and condition-dependent *Escherichia coli* proteome. Nat. Biotechnol. 2016; 34:104–110. 26641532 10.1038/nbt.3418 PMC4888949

[46] Qi F. , Motz M., Jung K., Lassak J., Frishman D Evolutionary analysis of polyproline motifs in *Escherichia coli* reveals their regulatory role in translation. PLoS Comput. Biol. 2018; 14:e1005987. 29389943 10.1371/journal.pcbi.1005987 PMC5811046

[47] Huter P. , Arenz S., Bock L.V., Graf M., Frister J.O., Heuer A., Peil L., Starosta A.L., Wohlgemuth I., Peske F. et al. Structural basis for polyproline-mediated ribosome stalling and rescue by the translation elongation factor EF-P. Mol. Cell. 2017; 68:515–527. 29100052 10.1016/j.molcel.2017.10.014

[48] Peil L. , Starosta A.L., Lassak J., Atkinson G.C., Virumäe K., Spitzer M., Tenson T., Jung K., Remme J., Wilson D.N. Distinct XPPX sequence motifs induce ribosome stalling, which is rescued by the translation elongation factor EF-P. Proc. Natl. Acad. Sci. U.S.A. 2013; 110:15265–15270. 24003132 10.1073/pnas.1310642110 PMC3780873

[49] Starosta A.L. , Lassak J., Peil L., Atkinson G.C., Virumäe K., Tenson T., Remme J., Jung K., Wilson D.N. Translational stalling at polyproline stretches is modulated by the sequence context upstream of the stall site. Nucleic Acids Res. 2014; 42:10711–10719. 25143529 10.1093/nar/gku768 PMC4176338

[50] Woolstenhulme C.J. , Guydosh N.R., Green R., Buskirk A.R. High-precision analysis of translational pausing by ribosome profiling in bacteria lacking EFP. Cell Rep. 2015; 11:13–21. 25843707 10.1016/j.celrep.2015.03.014 PMC4835038

[51] Gong F. , Ito K., Nakamura Y., Yanofsky C. The mechanism of tryptophan induction of tryptophanase operon expression: tryptophan inhibits release factor-mediated cleavage of TnaC-peptidyl-tRNA^Pro^. Proc. Natl. Acad. Sci. U.S.A. 2001; 98:8997–9001. 11470925 10.1073/pnas.171299298 PMC55362

[52] Nakatogawa H. , Ito K. Secretion monitor, secM, undergoes self-translation arrest in the cytosol. Mol. Cell. 2001; 7:185–192. 11172723 10.1016/s1097-2765(01)00166-6

[53] Chadani Y. , Niwa T., Izumi T., Sugata N., Nagao A., Suzuki T., Chiba S., Ito K., Taguchi H. Intrinsic ribosome destabilization underlies translation and provides an organism with a strategy of environmental sensing. Mol. Cell. 2017; 68:528–539. 29100053 10.1016/j.molcel.2017.10.020

[54] Keiler K.C. , Waller P.R.H., Sauer R.T. Role of a peptide tagging system in degradation of proteins synthesized from damaged messenger RNA. Science. 1996; 271:990–993. 8584937 10.1126/science.271.5251.990

[55] Karzai A.W. , Susskind M.M., Sauer R.T. SmpB, a unique RNA-binding protein essential for the peptide-tagging activity of SsrA (tmRNA). EMBO J. 1999; 18:3793–3799. 10393194 10.1093/emboj/18.13.3793 PMC1171456

[56] Chadani Y. , Ono K., Ozawa S., Takahashi Y., Takai K., Nanamiya H., Tozawa Y., Kutsukake K., Abo T. Ribosome rescue by *Escherichia coli* ArfA (YhdL) in the absence of trans-translation system. Mol. Microbiol. 2010; 78:796–808. 21062370 10.1111/j.1365-2958.2010.07375.x

[57] Chadani Y. , Ono K., Kutsukake K., Abo T. *Escherichia coli* YaeJ protein mediates a novel ribosome-rescue pathway distinct from SsrA- and ArfA-mediated pathways. Mol. Microbiol. 2011; 80:772–785. 21418110 10.1111/j.1365-2958.2011.07607.x

[58] Dougan D.A. , Truscott K.N., Zeth K. The bacterial N-end rule pathway: expect the unexpected. Mol. Microbiol. 2010; 76:545–558. 20374493 10.1111/j.1365-2958.2010.07120.x

