# Supplementary material for: Drop-off-reinitiation triggered by EF-G-driven mistranslocation and its alleviation by EF-P
Source: Nucleic Acids Res. 2022 Feb 21;50(5):2736–53. doi: 10.1093/nar/gkac068 (PMC8934632; doi:10.1093/nar/gkac068)
Supplement: gkac068_Supplemental_Files [file gkac068_supplemental_files.zip › 220113supplementary figure.pdf]

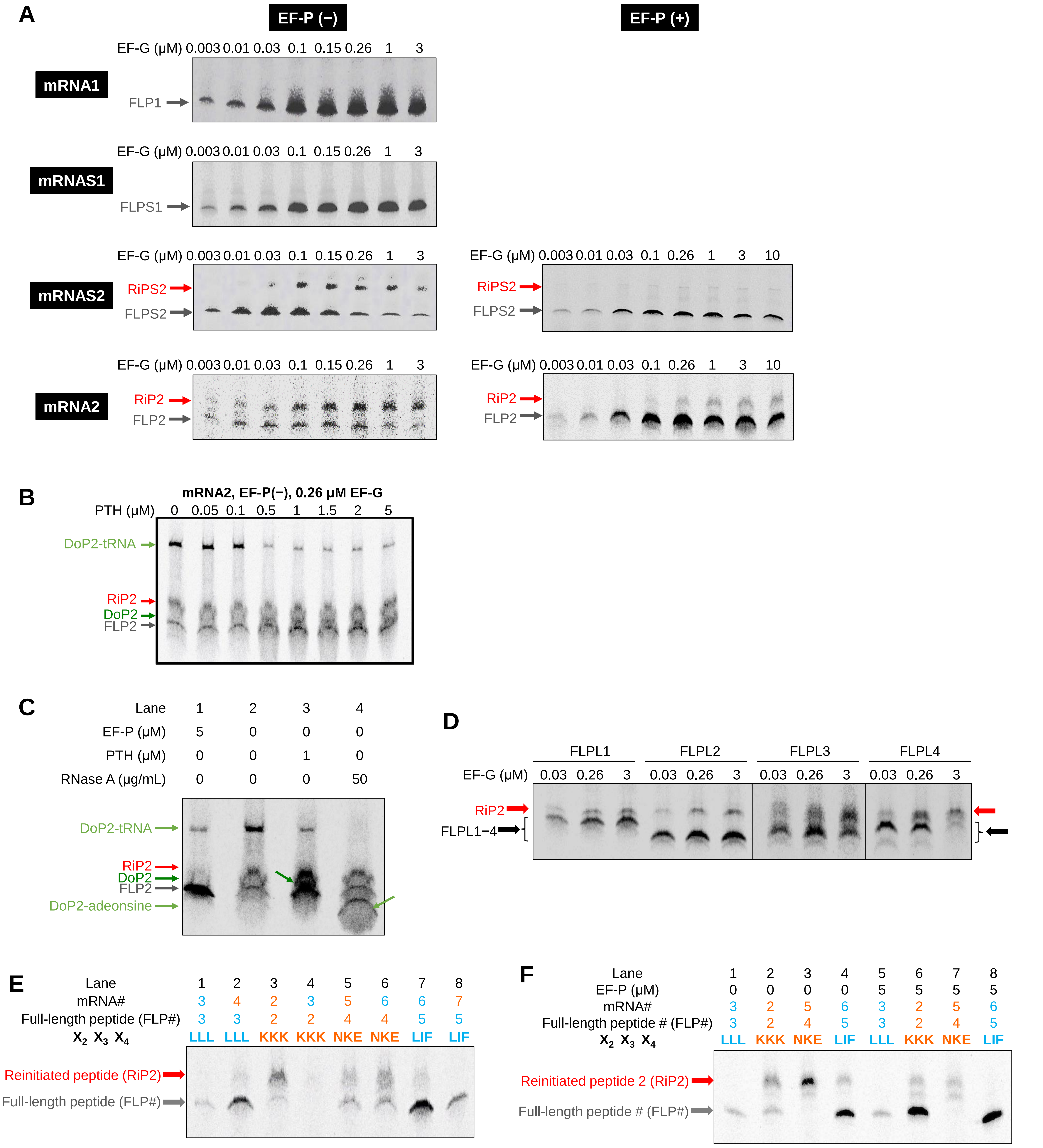

Supplementary Figure S1. Tricine-SDS-PAGE of the expressed peptides. (A) Titration of EF-G concentration in translation of mRNA1, mRNAS1, mRNAS2, and mRNA2 in the absence and presence of EF-P. Expressed peptides were radioisotope-labelled by [^{14}C]-Asp in the C-terminal FLAG sequences, separated by tricine-SDS-PAGE, and quantified by autoradiography. See also Figure 1A for the quantification results. **(B)** Titration of PTH concentration in hydrolysis of peptidyl-tRNAs generated by the translation of mRNA2 in the absence of EF-P and in the presence of 0.26 μM EF-G. Peptides were radioisotope-labelled with [^{14}C]-Lys to detect FLP2 (grey arrow), DoP2-tRNA (light green arrow), RiP2 (red arrow), and DoP2 (green arrow). **(C)** Cleavage of a peptidyl-tRNA by PTH and RNase A. mRNA2 was translated in the presence of [^{14}C]-Lys. FLP2 (grey arrow), DoP2-tRNA or DoP2-adenosine (light green arrow), RiP2 (red arrow), and DoP2 (green arrow). **(D)** Expression of FLPL1−4 in titration of EF-G concentration in the absence of EF-P. FLPL peptides (grey arrow) and RiP2 (red arrow) were radioisotope-labelled by the [^{14}C]-Asp in the FLAG sequences, separated by tricine-SDS-PAGE, and quantified by autoradiography (See also Figure 4B for the quantification result). Tris(2-carboxyethyl)phosphine (TCEP) was added to the translation reaction mix to reduce the sulfhydlyl group of cysteine (C). **(E,F)** Expression of the four kinds of peptides (FLP2−5) from two different mRNAs using the canonical or reprogrammed genetic codes in the absence (E: lanes 1−8, F: lanes 1−4) or presence of EF-P (F: lanes 5−8). The peptides were radio-labelled with [^{14}C]-Asp and quantified by autoradiography after tricine-SDS-PAGE. See also Figure 5C, D for the quantification results.

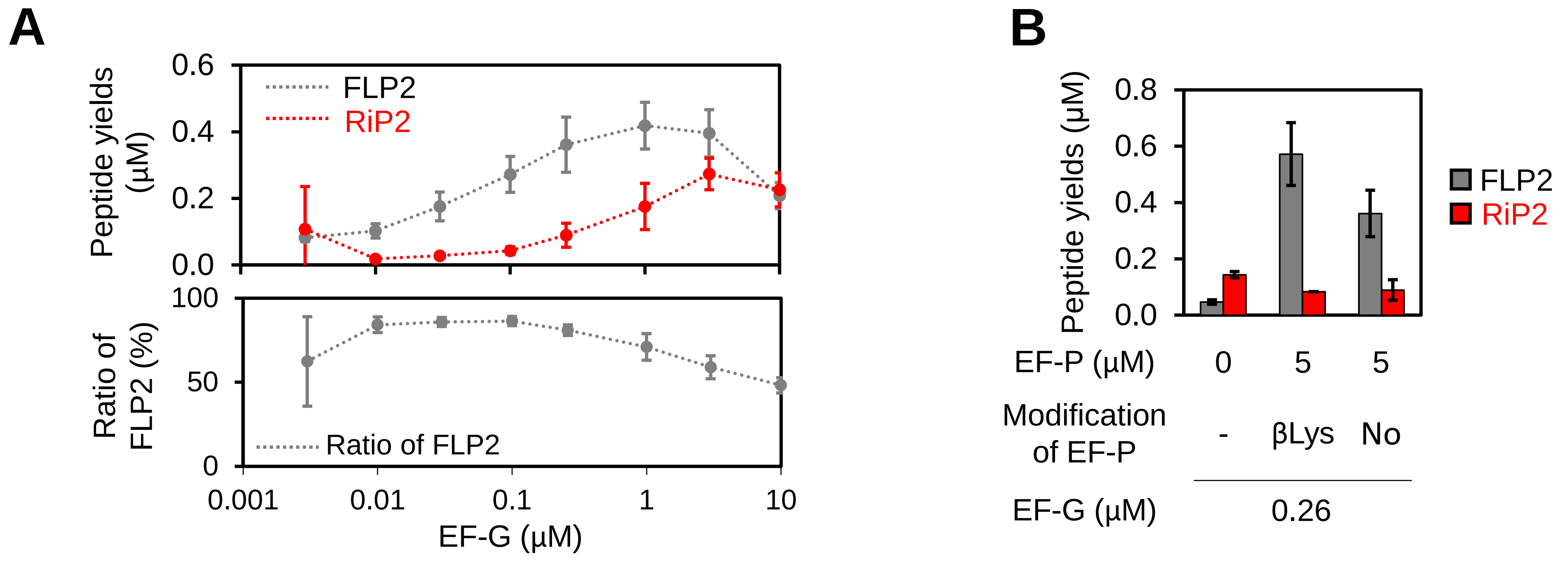

Supplementary Figure S2. Titration of EF-G concentration in the presence of unmodified EF-P in translation of mRNA2. (A) Yields of FLP2 and RiP2 in titration of EF-G in the presence of unmodified EF-P. Expressed peptides were radioisotope-labelled by [^{14}C]-Asp in the C-terminal FLAG sequences, separated by tricine-SDS-PAGE, and quantified by autoradiography. n = 3. **(B)** Comparison of peptide yields expressed without EF-P, with β-lysinylataed EF-P, or unmodified EF-P. Yields of FLP2 and RiP2 were quantified by autoradiography. n = 3.

A

mRNAM1:	5′ AUG^{Ini}	AAG	AAG	AAG	AUG^{Elo}	(flag)	UAA 3′
Full-length proteinogenic peptide M1 (FLPM1(**fM**, **M**)):	**fM**	**K**	**K**	**K**	**M**	**Flag**	**Stop**
Full-length deformylated peptide M1 (FLPM1(**M**, **M**)):	**M**	**K**	**K**	**K**	**M**	**Flag**	**Stop**
Full-length N-biotinylated peptide M1 (FLPM1(^{Bio}**F**, **M**)):	^{Bio}**F**	**K**	**K**	**K**	**M**	**Flag**	**Stop**

B

Lane	1	2	3	4	5
0.5 mM Free Met	−	+		−	+
10-HCO-H4folate	−	+	−		
aa assigned in AUG^{ini} by HOW	−	fM MRS	M MRS	^{Bio}F eFx	^{Bio}F eFx
aa assigned in AUG^{Elo} by HOW	−	M MRS	M MRS	M dFx	M MRS

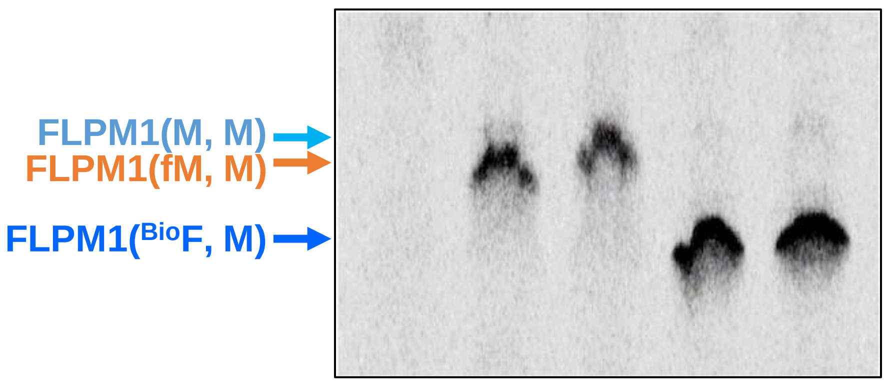

C

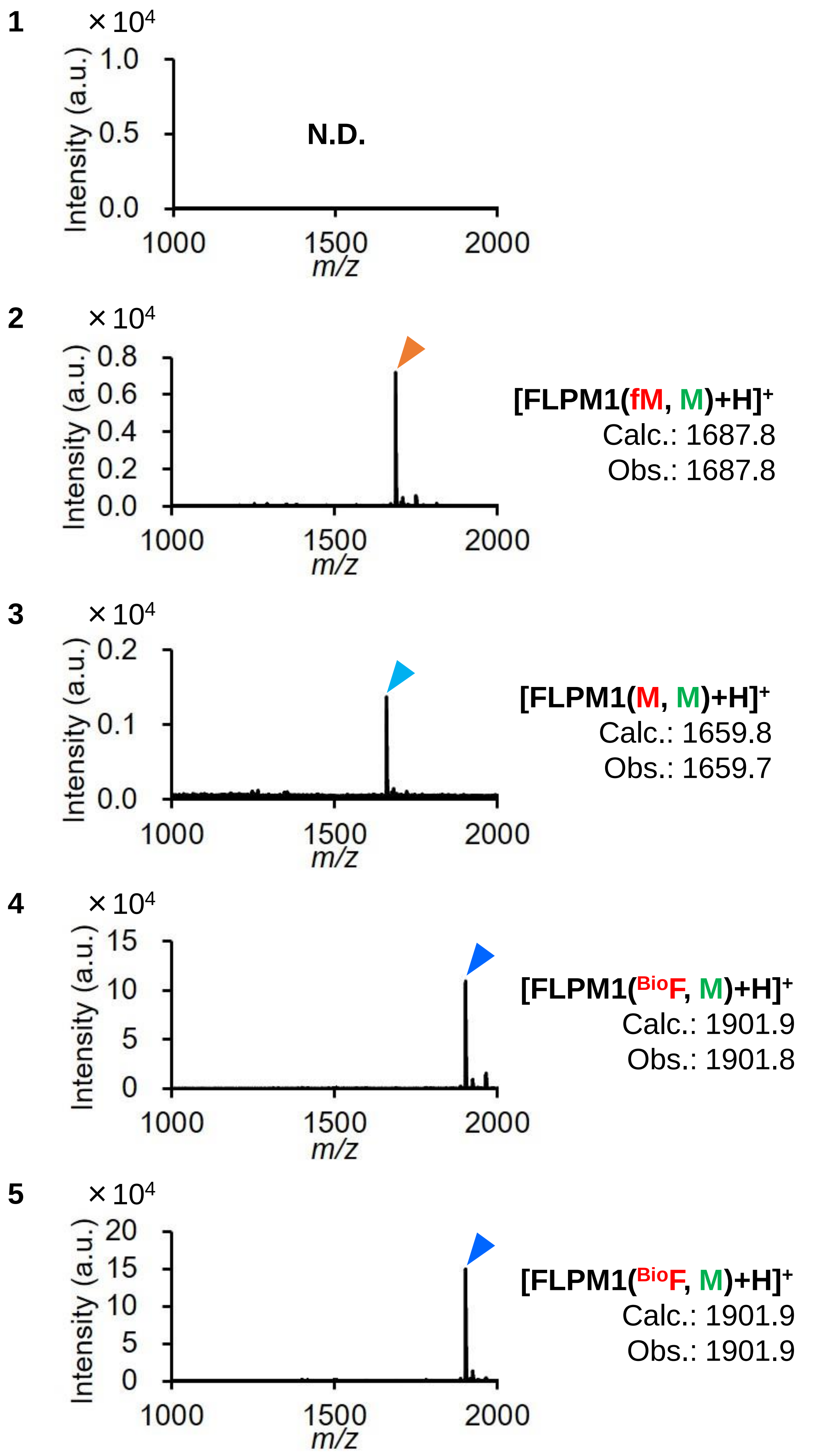

Supplementary Figure S3. Coding different amino acids in the initiator and elongator AUG codons. (A) An mRNA sequence and the corresponding peptide sequences used in this analysis. **(B)** Assignment of different amino acids to the initiator AUG codon (AUG^{Ini}) and the elongator AUG codon (AUG^{Elo}). mRNAM1 having both AUG^{Ini} and AUG^{Elo} was expressed without Met and 10-HCO-H4folate for formylation of M-tRNA^{fMet} (control for no expression, Lane 1), with both of Met and 10-HCO-H4folate in the presence of MRS (control for coding fM and M into AUG^{Ini} and AUG^{Elo}, Lane 2), with Met but without 10-HCO-H4folate (control for coding M into both of AUG^{Ini} and AUG^{Elo}, Lane 3), with pre-charged ^{Bio}F-tRNA^{fMet} and M-tRNA^{AsnE2} (coding ^{Bio}F and M into AUG^{Ini} and AUG^{Elo}, Lane 4), and pre-charged ^{Bio}F-tRNA^{fMet} and Met charged onto tRNA^{Met} *in situ* (coding ^{Bio}F and M into AUG^{Ini} and AUG^{Elo}, Lane 5). MRS: methionyl-tRNA synthetase, eFx: enhanced Flexizyme, dFx: dinitro Flexizyme. **(C)** Identification expressed peptides by MALDI-TOF MS. The same nomenclature as in (B).

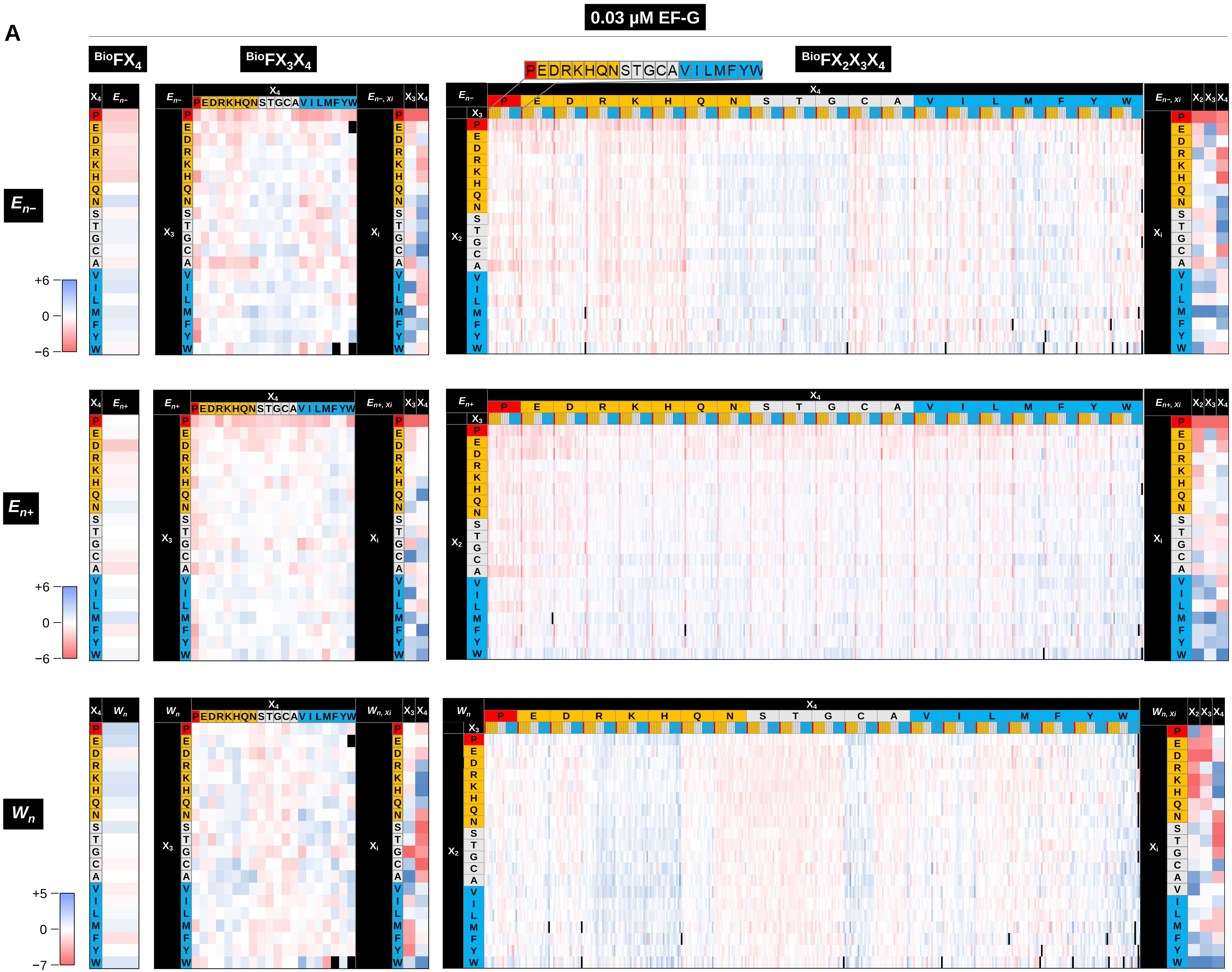

Supplementary Figure S4. Profiling of nascent peptides $^{Bio}FX_4$, $^{Bio}FX_3X_4$, and $^{Bio}FX_2X_3X_4$. (A,B,C) Heat maps of E_{n-} and $E_{n-,\ xi}$ (top), E_{n+} and $E_{n+,\ xi}$ (middle), and W_n. and $W_{n,\ xi}$ (bottom) in translation of mRNA library 1 ($^{Bio}FX_4$), library 2 ($^{Bio}FX_3X_4$), and library 3 ($^{Bio}FX_2X_3X_4$) in the presence of 0.03 µM (A), 0.26 µM (B), or 10 µM (C) of EF-G. $W_n = E_{n+} - E_{n-}$. X_2, X_3, and X_4 are one of the 20 proteinogenic amino acids. $E_{n-,\ xi}$, $E_{n+,\ xi}$, and $W_{n,\ xi}$ are average E_{n-}, E_{n+}, and $W_{n,}$ values, respectively, in which an amino acid X_i (i = 2, 3 or 4) is fixed to one of the 20 proteinogenic amino acids. Peptide sequences which were not detected by the next generation sequencing were filled with black. **(D,E,F)** Heat maps of E_{n-} and $E_{n-,\ xi}$ (top), E_{n+} and $E_{n+,\ xi}$ (middle), and W_n. and $W_{n,\ xi}$ (bottom) in translation of mRNA library 1 ($^{Bio}FX_4$), library 2 ($^{Bio}FX_3X_4$), and library 3 ($^{Bio}FX_2X_3X_4$), where the Pro-Pro-Pro sequence was substituted with Gly-Gly-Gly. The concentration of EF-G was 0.03 µM (D), 0.26 µM (E), or 10 µM (F).

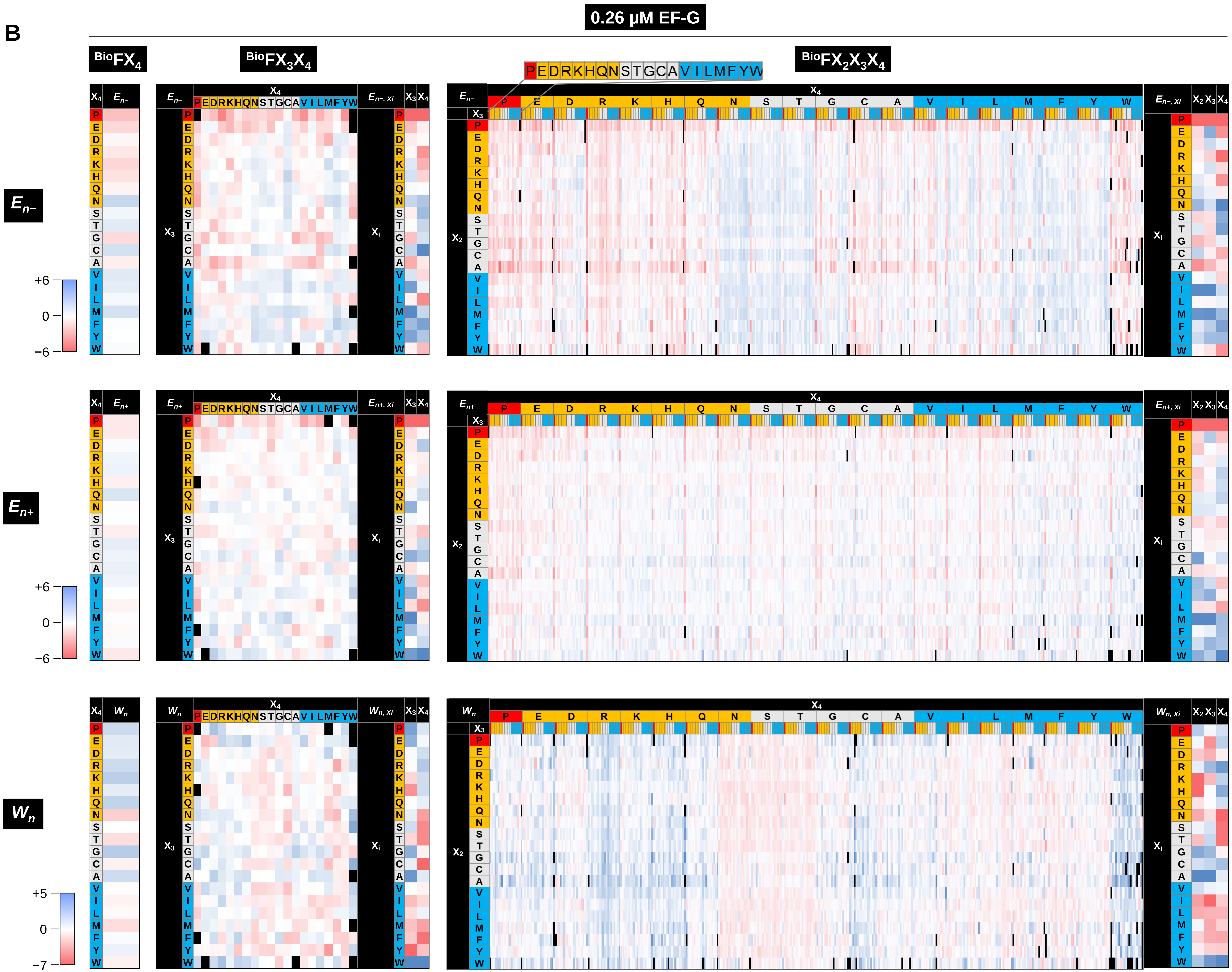

Supplementary Figure S4, continued.

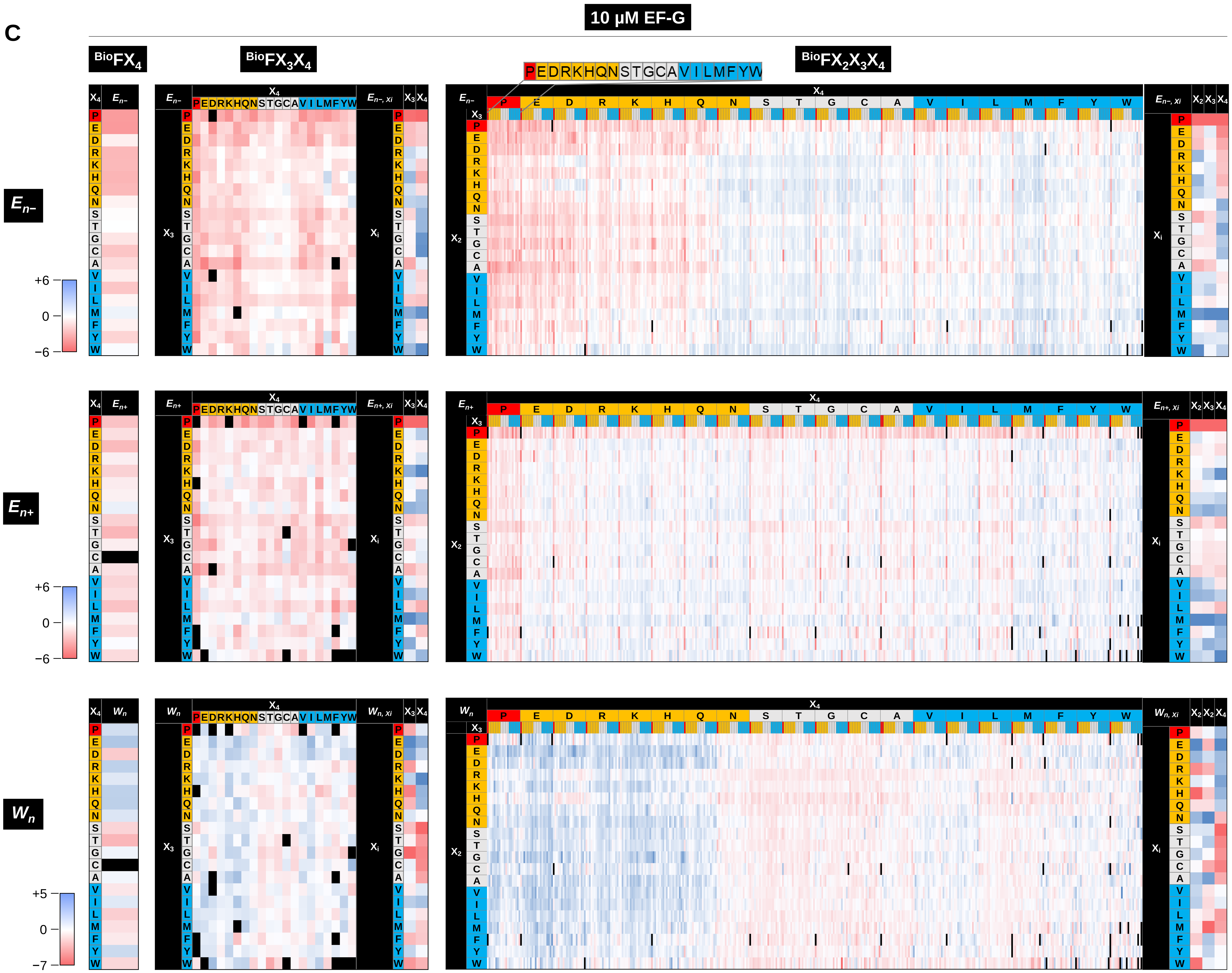

Supplementary Figure S4, continued.

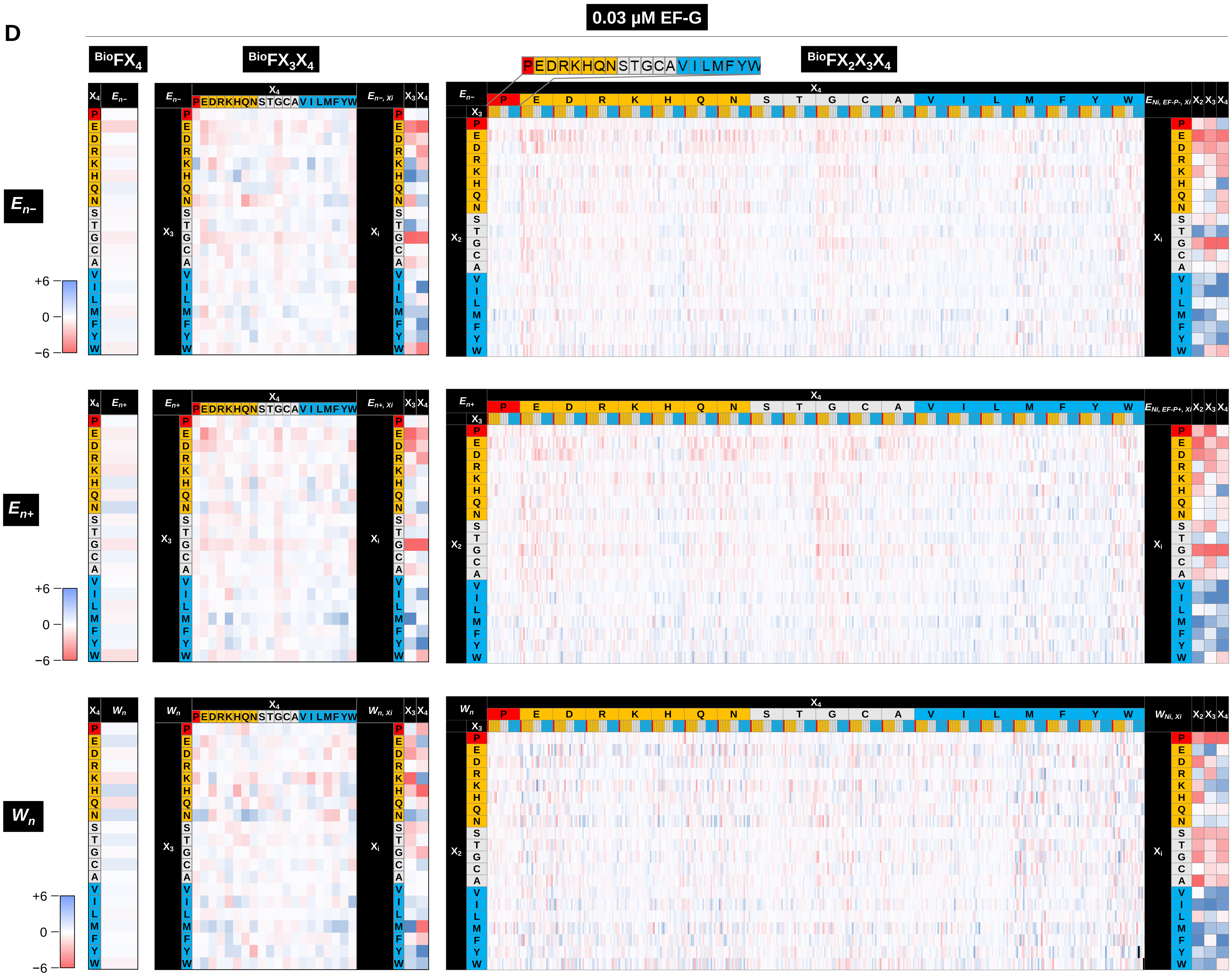

Supplementary Figure S4, continued.

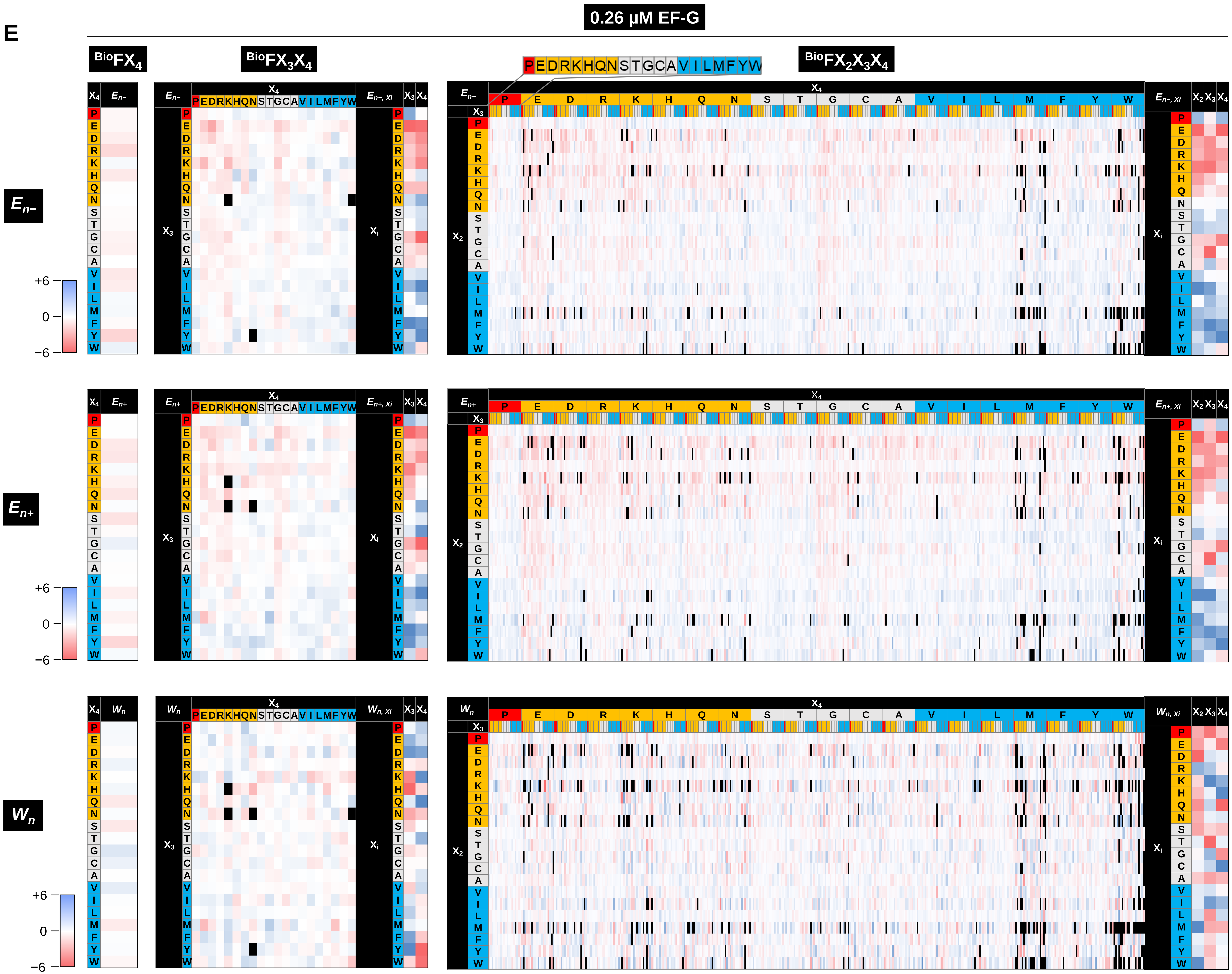

Supplementary Figure S4, continued.

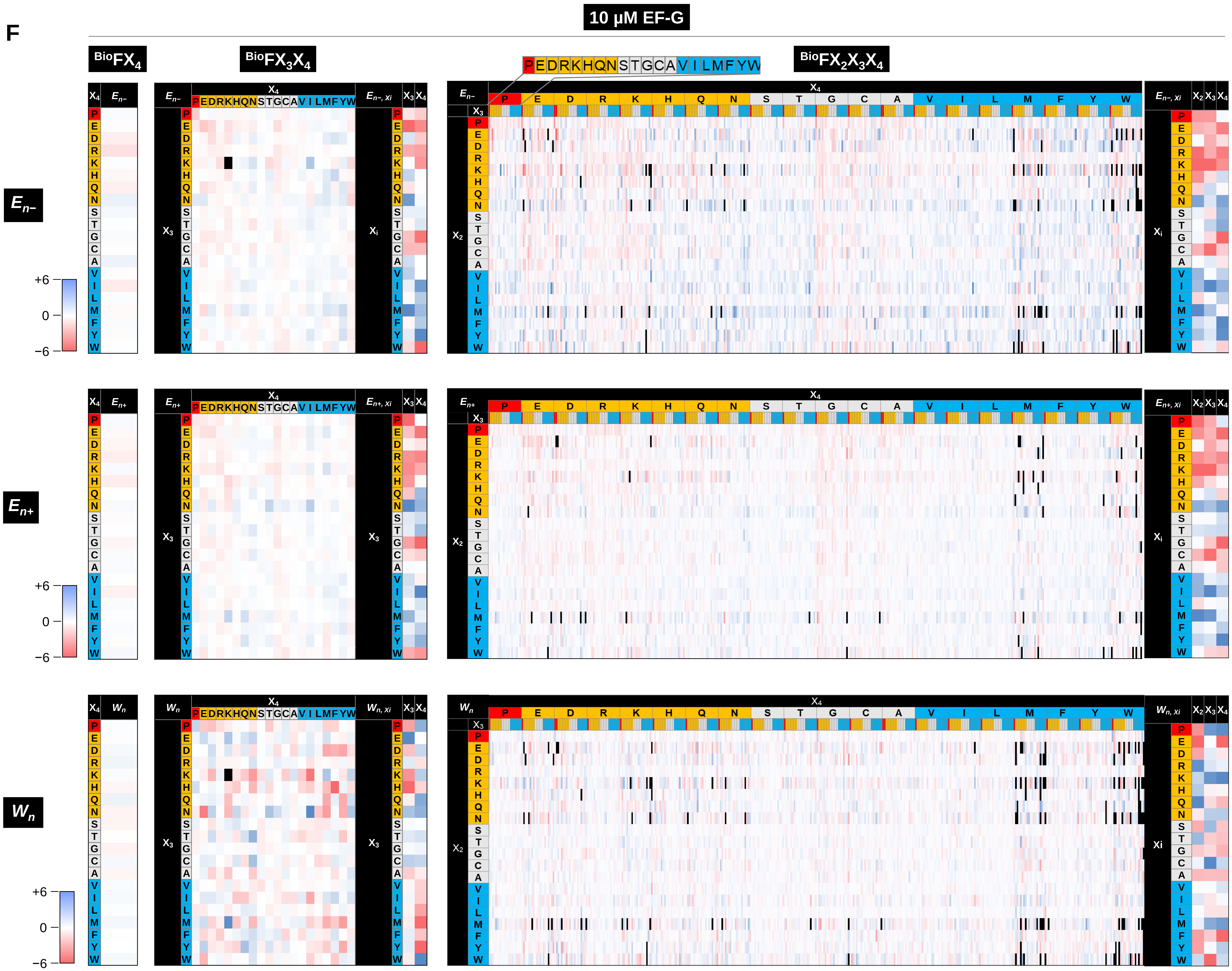

Supplementary Figure S4, continued.

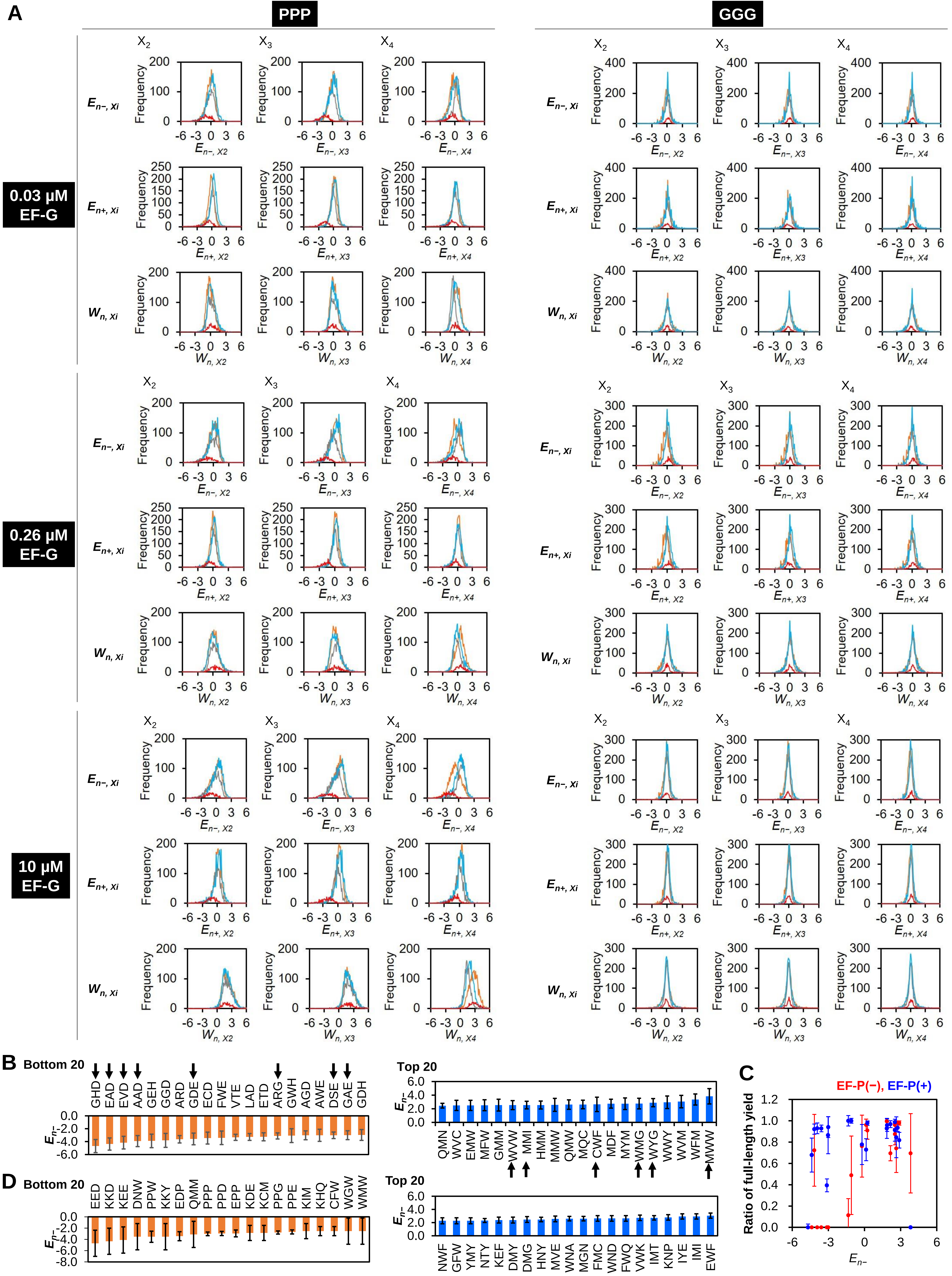

Supplementary Figure S5. Histograms of nascent chain profiling data in incorporation of three consecutive prolines or glycines. (A) Histograms of $E_{n-,\,Xi}$, $E_{n+,\,Xi}$, and $W_{n,\,Xi}$ (i = 2, 3, or 4) in incorporation of three consecutive prolines (PPP) or glycines (GGG) with 0.03, 0.26, and 10 µM EF-G. Histograms were drawn in red when X_i is proline (P), in orange when X_i is polar amino acid (E, D, R, K, H, Q, N), in grey when X_i is small amino acid (S, T, G, C, A), or in sky-blue when X_i is hydrophobic amino acid (V, I, L, M, F, Y, W). **(B)** $X_2X_3X_4$ sequences with the bottom and top 20 E_{n-} values in incorporation of PPP with 10 µM EF-G in the absence of EF-P. Error bar: 95% confident interval. *In vitro* expressed sequences are indicated by arrows. **(C)** Comparison of the sequencing data with LC-ESI MS quantification. Three prolines were consecutively incorporated into representative nascent peptides *in vitro* in the absence or presence of 5 µM EF-P. The expression levels of peptides estimated by LC-ESI MS were converted into the ratio of full-length peptide yield [FLP / (FLP + RiP)]. Red: In the presence of EF-P, Blue: In the absence of EF-P. **(D)** $X_2X_3X_4$ sequences with the bottom and top 20 E_{n-} values in incorporation of GGG with 10 µM EF-G in the absence of EF-P. Error bar: 95% confident interval. **(E)** Sequences of mRNAX# and the corresponding peptides, FLPX# and RiPX, bearing $X_2X_3X_4$. The $X_2X_3X_4$ sequences with high and low E_{n-} values (B, indicated by black arrows) were used for translation and LC-ESI MS analysis. **(F)** XICs of representative FLPX# and RiPX expressed *in vitro* with 10 µM EF-G in the absence of EF-P. The $X_2X_3X_4$ sequences are indicated in blue (high E_{n-} values) or orange (low E_{n-} values). Sky-blue XICs were extracted by the *m/z* values of respective full-length peptides and orange XICs by the *m/z* value of RiPX. Closed triangles indicate peaks corresponding to the respective full-length peptides and open triangle indicate peaks corresponding to RiPX. **(G)** XICs of representative FLPX# and RiPX expressed *in vitro* with 10 µM EF-G in the presence of 5 µM EF-P. RiPX was not detected in all cases.

E

mRNAX#:	AUG	NNN	NNN	NNN	CCG	CCG	CCG	AGC	GGU	AAC	(flag)	UAA
Full-length peptide X# (FLPX#):	BioF	X_2	X_3	X_4	P	P	P	S	G	N	FLAG	(Stop)
Reinitiated Peptide X (RiPX):							P	S	G	N	FLAG	

F

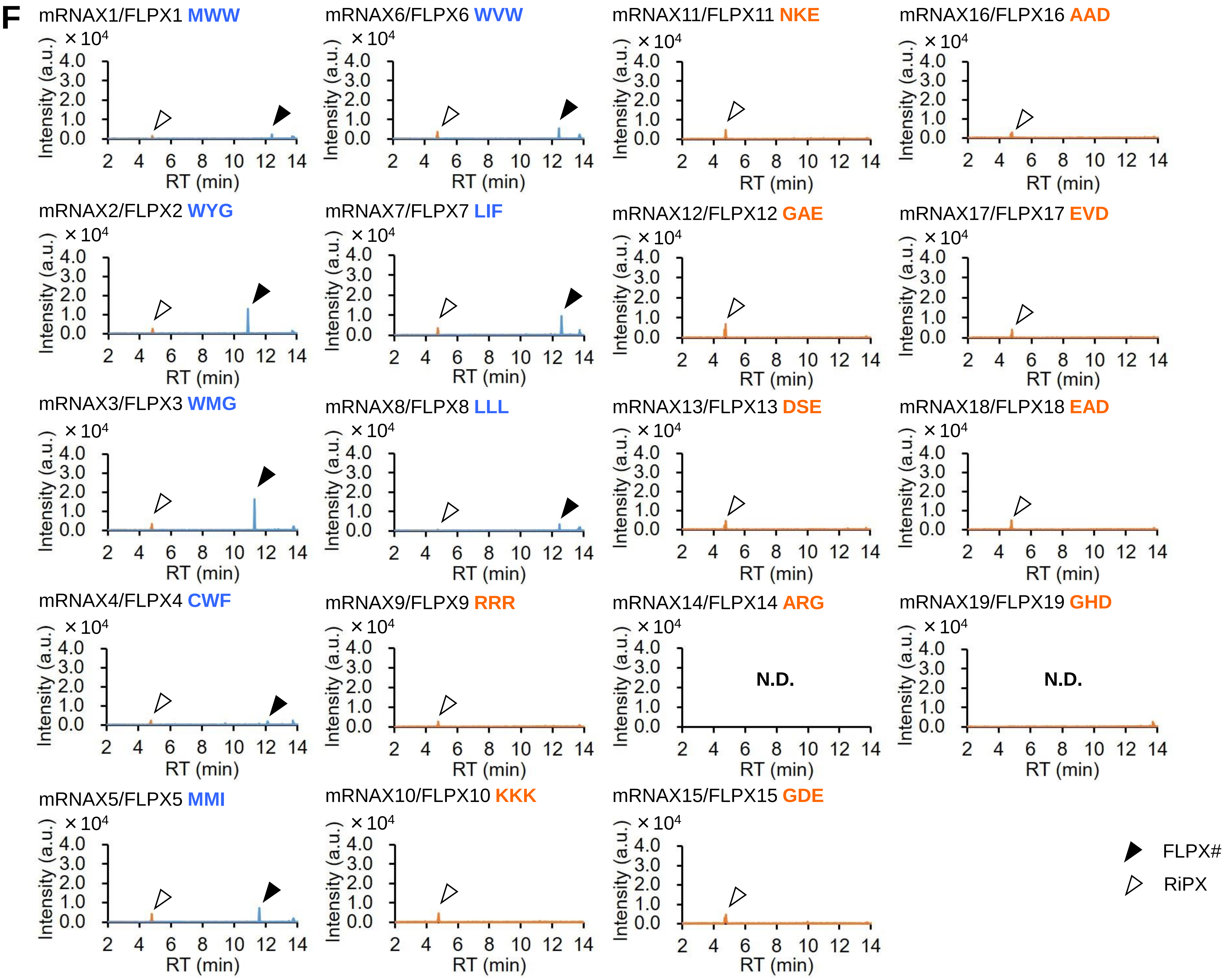

G

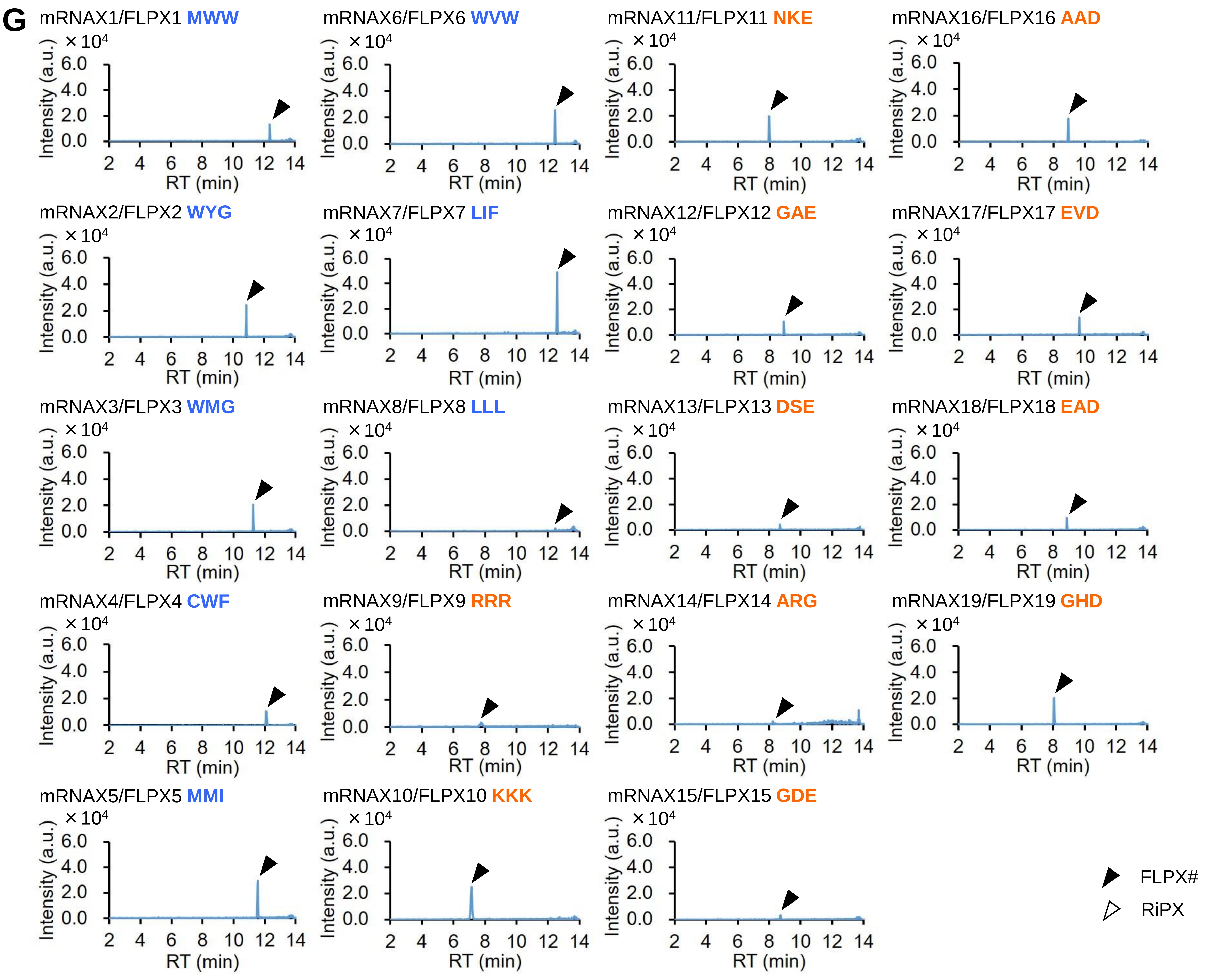

Supplementary Figure S5, continued.

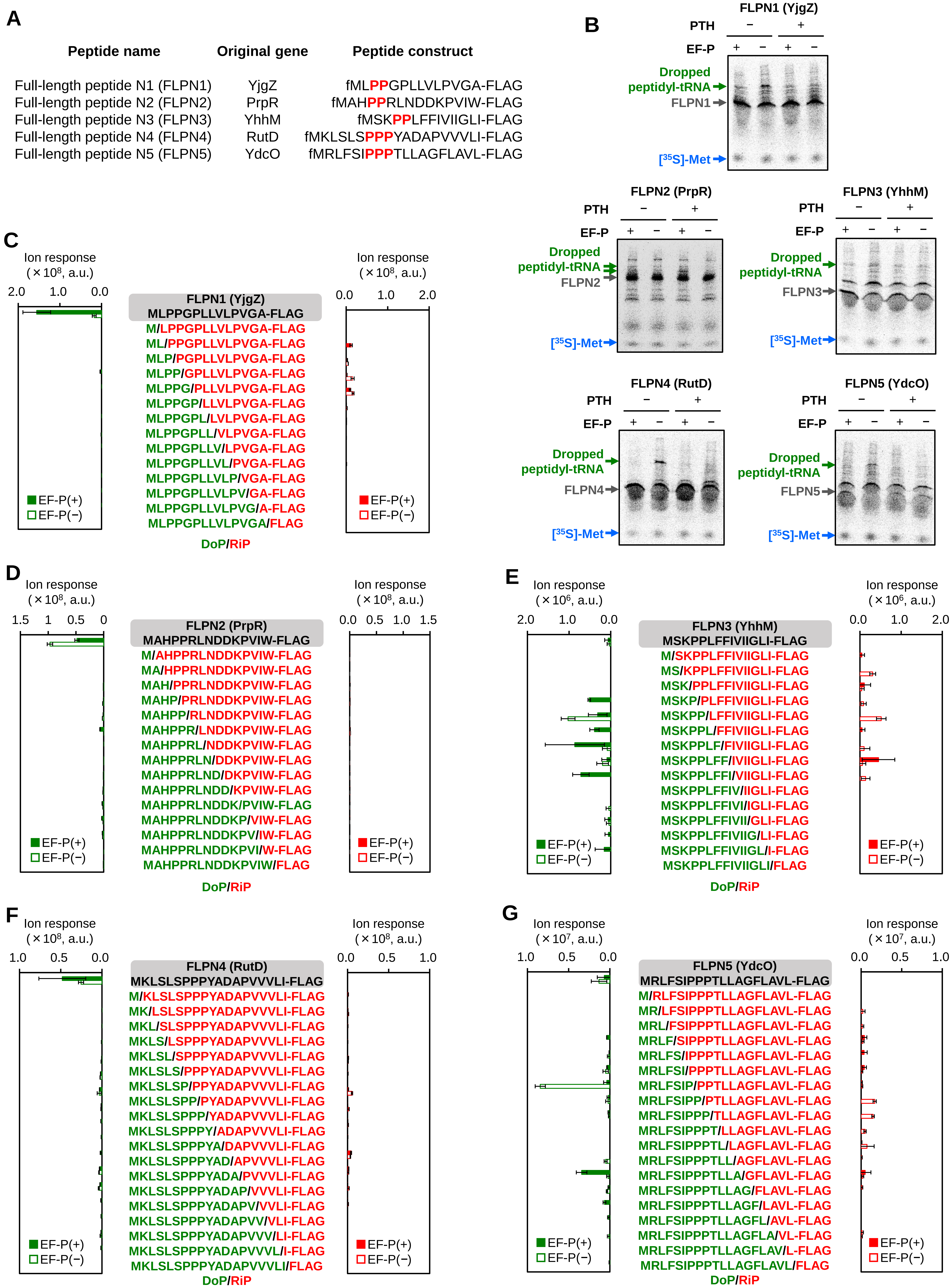

Supplementary Figure S6. ***In vitro*** **and in-cell expression of N-terminal region of polyproline-containing proteins. (A)** Peptide sequences derived from N-terminal region of *E. coli* proteins containing two or three consecutive prolines. FLAG-tag was added to the C-termini of peptides. **(B)** Detection of peptidyl-tRNA drop-off in *in vitro* expression of peptides FLPN 1–5. Peptides were N-terminally radioisotope-labelled by [35S]-Met in the presence and absence of 5 μM EF-P, treated with PTH, and visualized by autoradiography after tricine-SDS-PAGE. The bands of dropped peptidyl-tRNA were identified by comparing PTH(−)/EF-P(−) lanes and PTH(+)/EF-P(−) lanes. **(C-G)** LC-ESI MS identification and quantification of N-terminal regions of polyproline-containing proteins expressed *in vitro*. FLPN 1−5 were expressed *in vitro* in the absence (open bars) or presence (closed bars) of 5 μM EF-P, treated with PTH, and all their possible DoPs (green bars) and RiPs (red bars) were quantified. Grey: Full-length peptides. Standard deviation of three independent experiments are shown. **(H-U)** LC-ESI MS/MS sequencing of N-terminal fragments of YhhM, YhhM-fh, expressed in *E. coli* in the absence (H−N) or presence (O−U) of EF-P and digested by Lys-N. Deconvoluted LC-ESI MS/MS spectra of FLP (fM, M) at RT = 10.95 min (H, O), FLP (fM, MOx) at RT = 10.62 min (I, P), FLP (fMOx, M) at RT = 10.77 min (J, Q), FLP (fMOx, MOx) at RT = 10.41 min (K, R), RiPΔN1 at RT = 10.96 min (L, S), RiPΔ2 at RT = 10.86 min (M) or 10.89 min (T), and RiPΔN3 at RT = 10.72 min (N) or 10.74 min (U). Green peaks are corresponding to daughter y ions and orange peaks corresponding to daughter b ions.

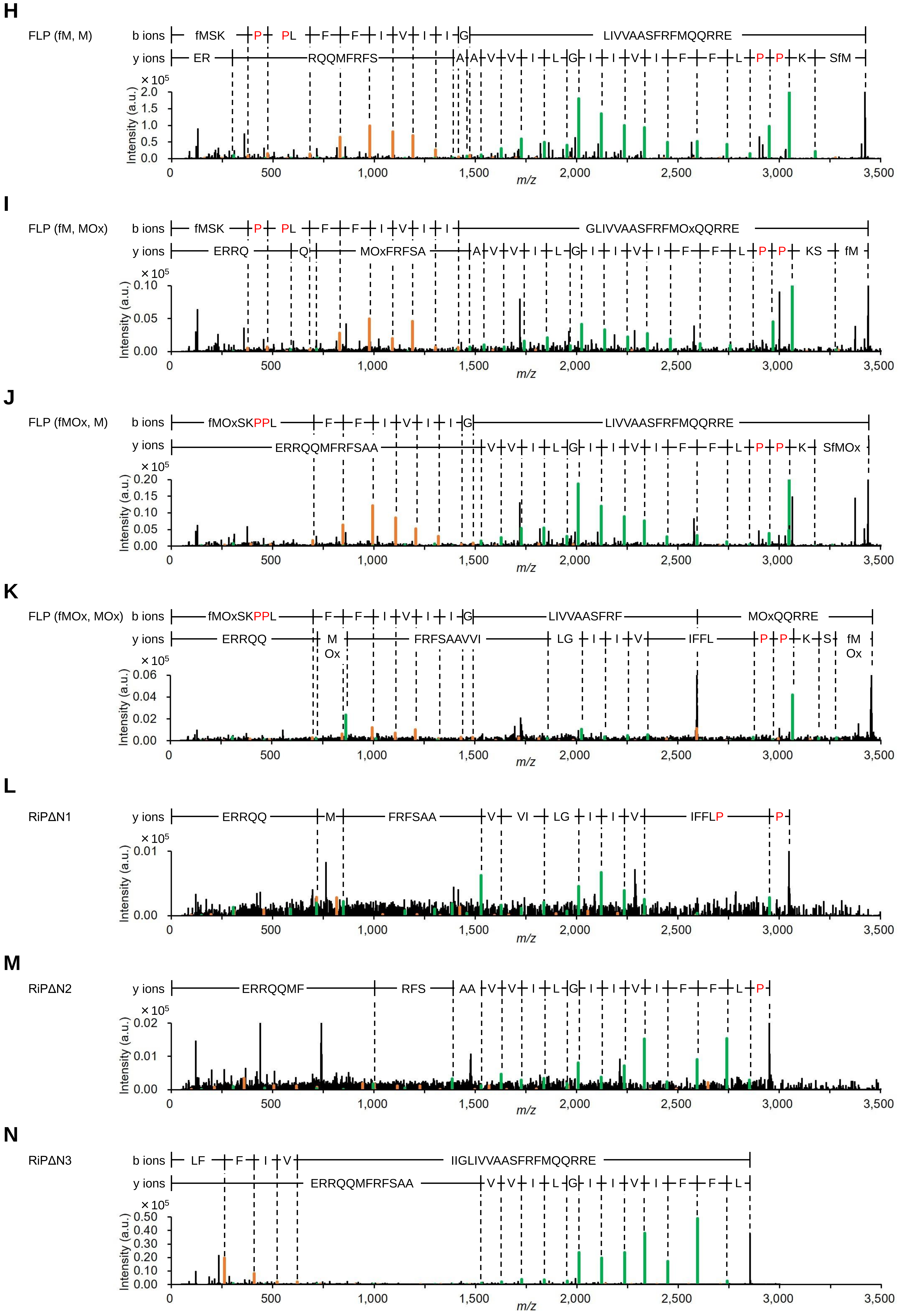

Supplementary Figure S6, continued.

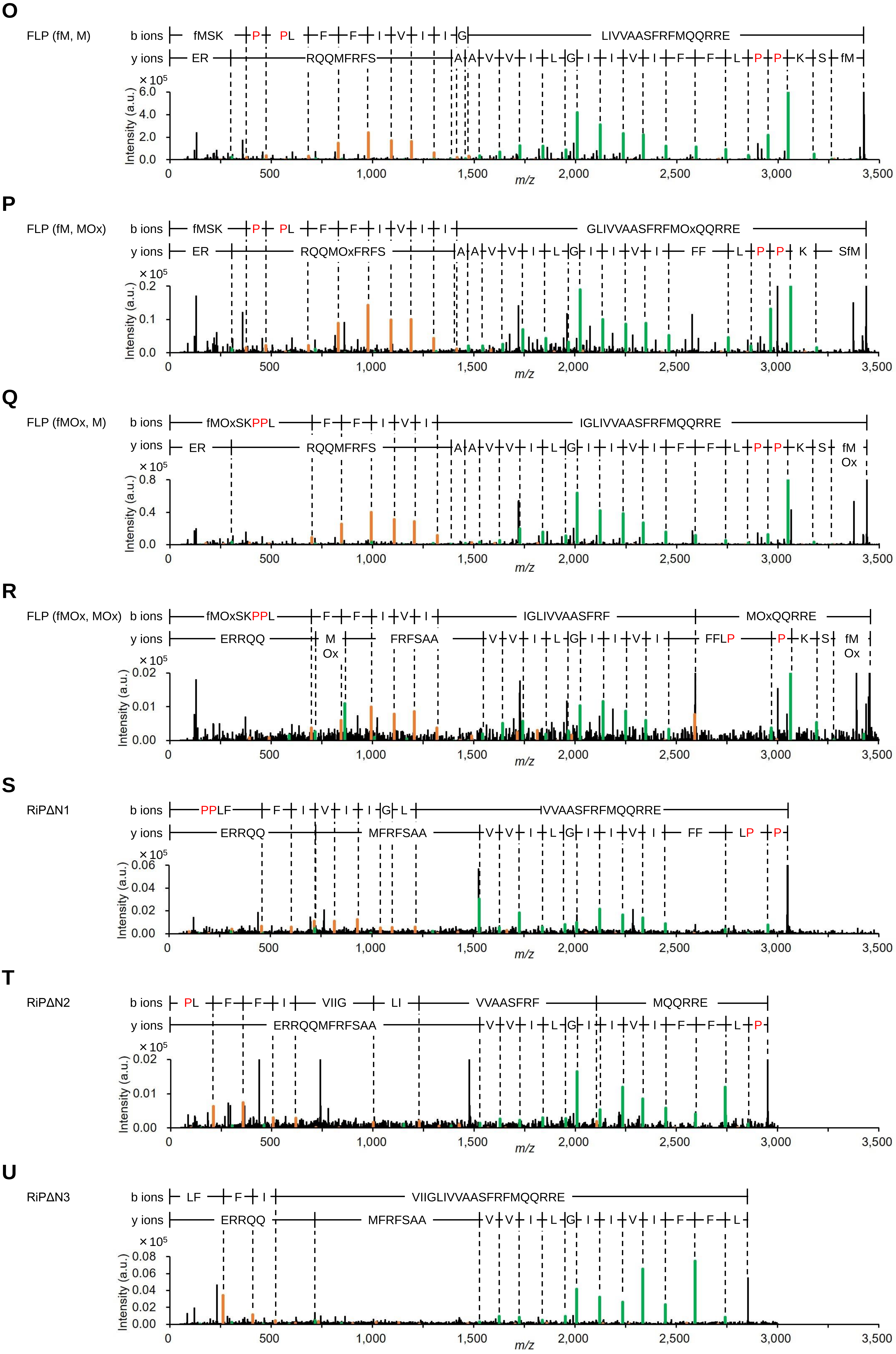

Supplementary Figure S6, continued.

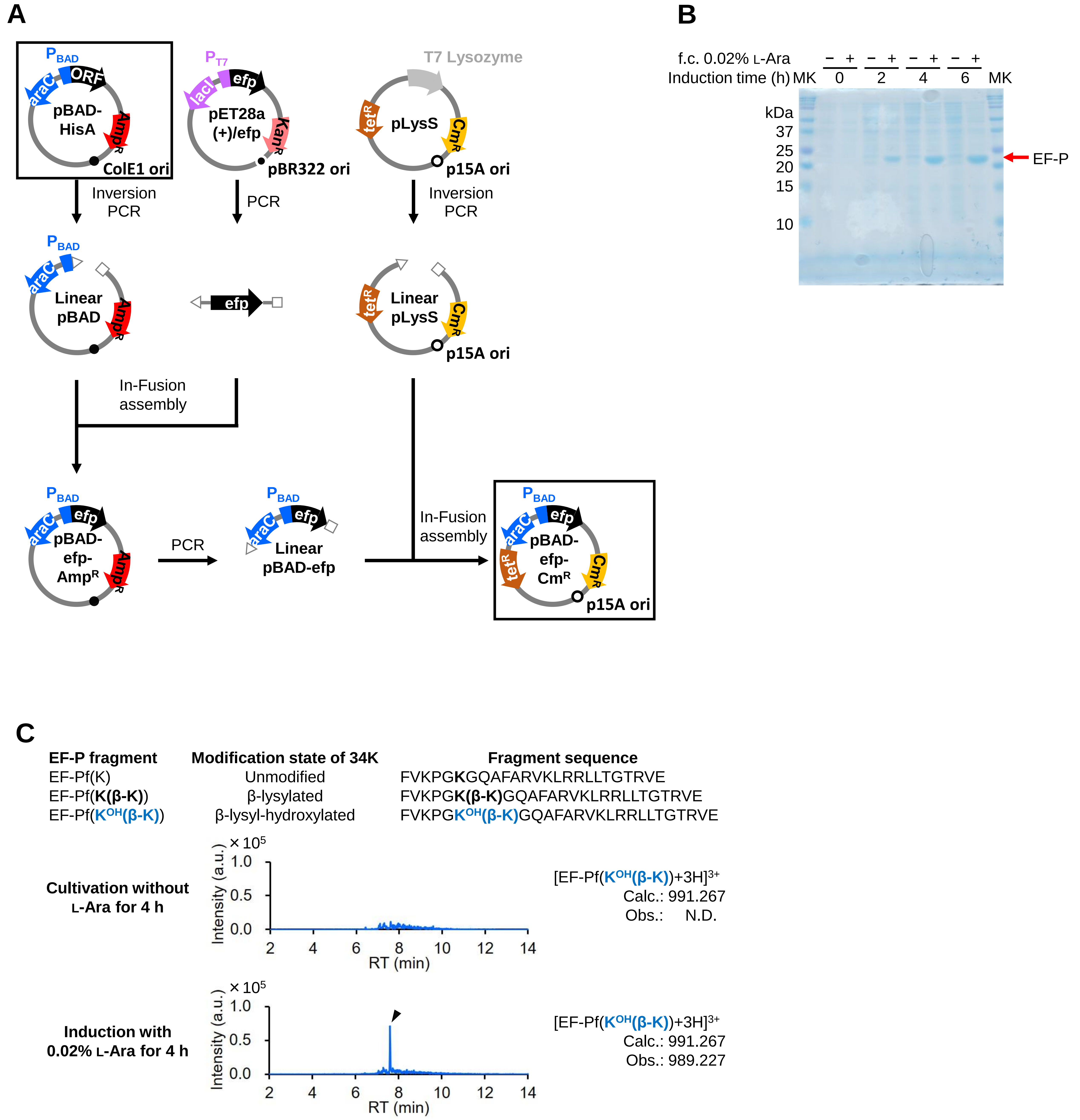

Supplementary Figure S7. Preparation of a plasmid coding EF-P and confirmation of post-translational modification state of EF-P complemented in *E. coli* Δ*efp* using the plasmid. (A) Preparation procedure of a plasmid coding EF-P. *efp* gene was cloned into a modified pBAD-HisA plasmid (pBAD-*efp*-CmR-p15a ori) and co-expressed with a protein containing consecutive prolines encoded in another pBAD-HisA plasmid derivative. **(B)** Expression of EF-P from pBAD-*efp*-CmR-p15a ori in *E. coli* Δ*efp* strain with or without induction by 0.02% L-arabinose (L-Ara) for 0, 2, 4, and 6 hours. **(C)** Confirmation of the post-translational modification of the complemented EF-P by endogenous EpmA/B/C modification enzymes. (Top) EF-P fragments with unmodified, β-lysylated, and β-lysyl-hydroxylated lysine (**K**, **K(β-K)**, and **K^{OH}(β-K)**) generated by GluC. (Middle) LC-ESI MS spectrum of *E. coli* Δ*efp* with pBAD-*efp*-CmR-p15a ori without induction, recovered by centrifuge, resuspended, and digested by Glu-C. (Bottom) LC-ESI MS spectrum of EF-P induced with 0.02% L-Ara for 4 hours, recovered by centrifuge, resuspended, and digested by Glu-C.

A

Fragment	Peptide	*z*	Calculated *m/z* of $[M+zH]^{z+}$	RT (min)
Lys-N3	KADNDMAPLQQ	2	615.795	5.801
Lys-N4	KLVVVSN	1	758.482	1.731
Lys-N5-6	KREKPINDRRSRQQEVTPAGTSIRYEASF	5	684.764	5.759
Lys-N6	KPINDRRSRQQEVTPAGTSIRYEASF	5	602.117	5.896
Lys-N7-8	KPQSGGMEQTFRLDAQQYHALTVGDKGTLSY	5	686.142	6.566
Lys-N8	KGTLSY	1	668.366	13.989
Lys-N9	KGTRFVSFVGEQDY	3	544.940	6.494

B

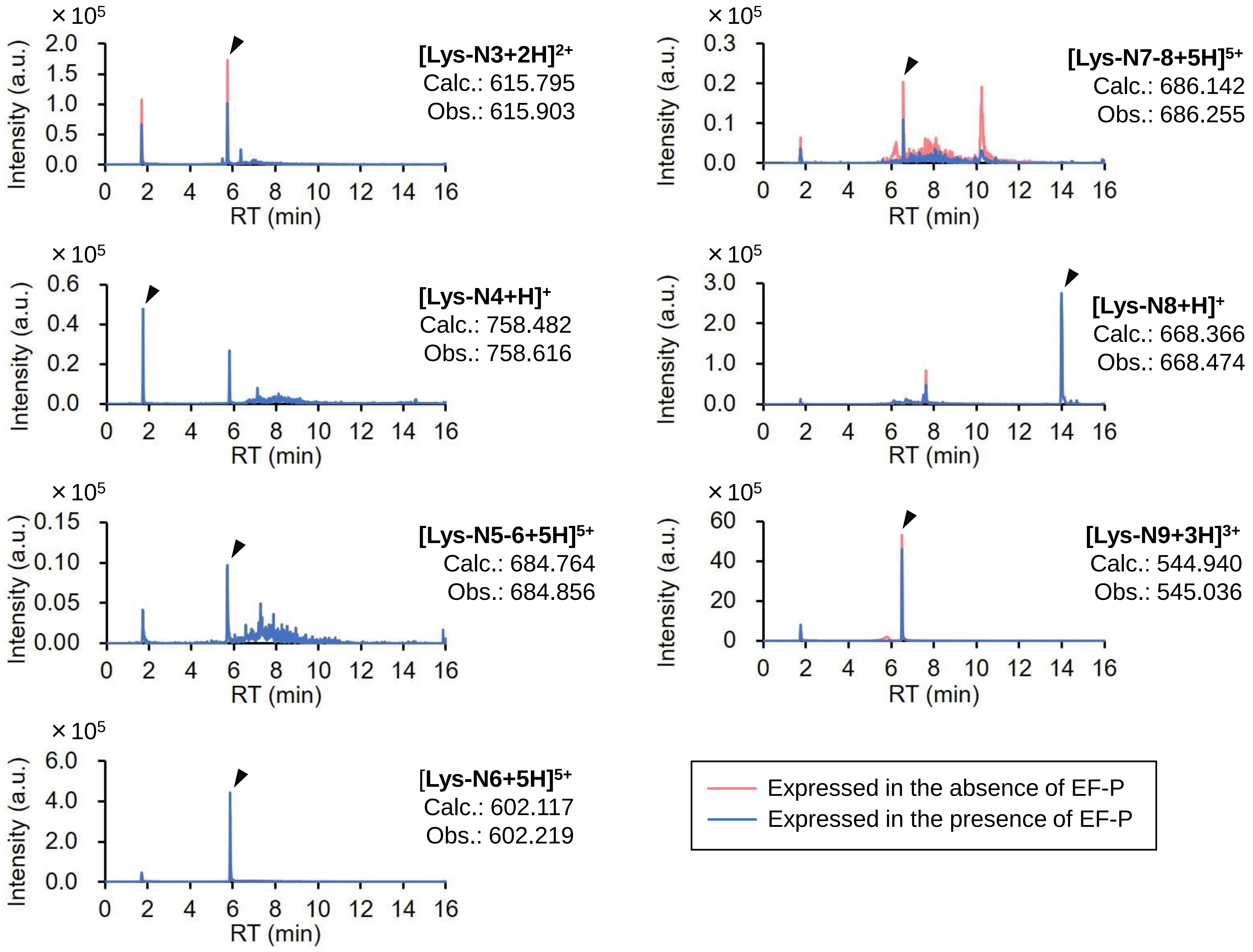

C

MS KPPLFFIVIIGLIVVAASFRFMQQRRE KADNDMAPLQQ KLVVVSN KRE KPINDRRSRQQEVTPAGTSIRYEASF
KPQSGGMEQTFRLDAQQYHALTVGD KGTLSY KGTRFVSFVGEQDY KDDDD KSSHHHHHH*

D

MS KPPLFFIVIIGLIVVAASFRFMQQRRE KADNDMAPLQQ KLVVVSN KRE KPINDRRSRQQEVTPAGTSIRYEASF
KPQSGGMEQTFRLDAQQYHALTVGD KGTLSY KGTRFVSFVGEQDY KDDDD KSSHHHHHH*

Supplementary Figure S8. Sequence coverage of YhhM-fh expressed in the absence and presence of EF-P detected by LC-ESI MS. (A) Identified peptide fragments derived from YhhM-fh digested by Lys-N except for the N-terminal fragments shown in Figure 7. Observed *z* values, calculated *m/z* values, and retention time (RT) in LC-ESI MS are shown. **(B)** XICs of peptide fragments derived from YhhM-fh expressed without coexpression of EF-P (Red) and coexpressed with EF-P (Blue) and digested by Lys-N. **(C)** Sequence coverage of identified peptide fragments derived from YhhM-fh expressed without coexpression of EF-P and digested by Lys-N. **(D)** Sequence coverage of identified peptide fragments derived from YhhM-fh coexpressed with EF-P and digested by Lys-N.
